# Author Correction: Pharmacological inhibition of PRMT7 links arginine monomethylation to the cellular stress response

**DOI:** 10.1038/s41467-020-16613-x

**Published:** 2020-05-26

**Authors:** Magdalena M. Szewczyk, Yoshinori Ishikawa, Shawna Organ, Nozomu Sakai, Fengling Li, Levon Halabelian, Suzanne Ackloo, Amber L. Couzens, Mohammad Eram, David Dilworth, Hideto Fukushi, Rachel Harding, Carlo C. dela Seña, Tsukasa Sugo, Kozo Hayashi, David McLeod, Carlos Zepeda, Ahmed Aman, Maria Sánchez-Osuna, Eric Bonneil, Shinji Takagi, Rima Al-Awar, Mike Tyers, Stephane Richard, Masayuki Takizawa, Anne-Claude Gingras, Cheryl H. Arrowsmith, Masoud Vedadi, Peter J. Brown, Hiroshi Nara, Dalia Barsyte-Lovejoy

**Affiliations:** 1 Structural Genomics Consortium, University of Toronto, Toronto, ON M5G 1L7 Canada; 2 Research, Takeda Pharmaceutical Company Limited, 26-1, Muraoka-Higashi 2-chome, Fujisawa, Kanagawa 251-8555 Japan; 3 Network Biology Collaborative Centre at the Lunenfeld-Tanenbaum Research Institute, 600 University Ave, Room 992, Toronto, ON M5G 1X5 Canada; 4 Drug Discovery Program, Ontario Institute for Cancer Research, Toronto, ON Canada; 5 Institute for Research in Immunology and Cancer (IRIC) University of Montreal, 2950 Chemin de Polytechnique, Montreal, QC H3T 1J4 Canada; 6 Department of Pharmacology and Toxicology, University of Toronto, Toronto, ON M5S 1A8 Canada; 7 Terry Fox Molecular Oncology Group and Bloomfield Center for Research on Aging, Lady Davis Institute for Medical Research and Departments of Oncology and Medicine, McGill University, Montreal, QC H3T 1E2 Canada; 8 Princess Margaret Cancer Centre and Department of Medical Biophysics, University of Toronto, Toronto, ON M5G 2M9 Canada; 9 Nature Research Center, Vilnius, Akademijos 2, Lithuania

**Keywords:** Transferases, Stress signalling, Methylation, Target validation

Correction to: *Nature Communications* 10.1038/s41467-020-16271-z, published online 14 May 2020.

The original version of this Article omitted the following from the Acknowledgements:

Research supported by SU2C Canada Cancer Stem Cell Dream Team Research Funding (SU2C-AACR-DT-19-15) provided by the Government of Canada through Genome Canada and the Canadian Institute of Health Research, with supplemental support from the Ontario Institute for Cancer Research, through funding provided by the Government of Ontario. Stand Up To Cancer Canada is a Canadian Registered Charity (Reg. # 80550 6730 RR0001). Research Funding is administered by the American Association for Cancer Research International—Canada, the Scientific Partner of SU2C Canada.

This has now been corrected in both the PDF and HTML versions of the Article.

