# Supplementary material for: Pharmacological inhibition of PRMT7 links arginine monomethylation to the cellular stress response
Source: Nat Commun. 2020 May 14;11:2396. doi: 10.1038/s41467-020-16271-z (PMC7224190; doi:10.1038/s41467-020-16271-z)
Supplement: Supplementary file 1 — Supplementary Information [file 41467_2020_16271_MOESM1_ESM.pdf]

Supplementary Information

Pharmacological inhibition of PRMT7 links arginine monomethylation to the cellular stress response

Magdalena M Szewczyk[1], Yoshinori Ishikawa[2*], Shawna Organ[1*], Nozomu Sakai[2*], Fengling Li[1*], Levon Halabelian[1], Suzanne Ackloo[1], Amber L Couzens[3], Mohammad Eram[1], David Dilworth[1], Hideto Fukushi[2], Rachel Harding[1], Carlo C dela Seña[1], Tsukasa Sugo[2], Kozo Hayashi[2], David McLeod[4], Carlos Zepeda[4], Ahmed Aman[4], Maria Sánchez-Osuna[5], Eric Bonneil[5], Shinji Takagi[2], Rima Al-Awar[4,8], Mike Tyers[5], Stephane Richard[6], Masayuki Takizawa[2], Anne-Claude Gingras[3], Cheryl H Arrowsmith[1,7], Masoud Vedadi[1,8], Peter J Brown[1], Hiroshi Nara[2#], Dalia Barsyte-Lovejoy[1,8,9#]

[1]Structural Genomics Consortium, University of Toronto, Toronto, Ontario, M5G 1L7, Canada

[2]Research, Takeda Pharmaceutical Company Limited, 26-1, Muraoka-Higashi 2-chome, Fujisawa, Kanagawa 251-8555, Japan

[3]Network Biology Collaborative Centre at the Lunenfeld-Tanenbaum Research Institute, 600 University Ave, Room 992, Toronto, Ontario, Canada, M5G 1X5

[4]Drug Discovery Program, Ontario Institute for Cancer Research, Toronto, Ontario, Canada

[5]Institute for Research in Immunology and Cancer (IRIC) University of Montreal, 2950 Chemin de Polytechnique, Montreal (QC) H3T 1J4, Canada

[6]Terry Fox Molecular Oncology Group and Bloomfield Center for Research on Aging, Lady Davis Institute for Medical Research and Departments of Oncology and Medicine, McGill University, Montreal, Quebec H3T 1E2, Canada

[7]Princess Margaret Cancer Centre and Department of Medical Biophysics, University of Toronto, Toronto, Ontario, M5G 2M9, Canada

[8]Department of Pharmacology and Toxicology, University of Toronto, Toronto, Ontario, M5S 1A8, Canada

[9]Nature Research Center, Vilnius, Akademijos 2, Lithuania

* Equal contribution
# Corresponding authors
Dalia Barsyte-Lovejoy, Structural Genomics Consortium, University of Toronto
Suite 700, MaRS South Tower, 101 College Street, Toronto, Ontario, M5G 1L7, Canada
Tel: +1-416-978-3867, Fax: +1-416-946-0588, d.barsyte@utoronto.ca
Hiroshi Nara, Research, Takeda Pharmaceutical Company Limited, 26-1, Muraoka-Higashi 2-chome, Fujisawa, Kanagawa 251-8555, Japan. nara@pharm.or.jp

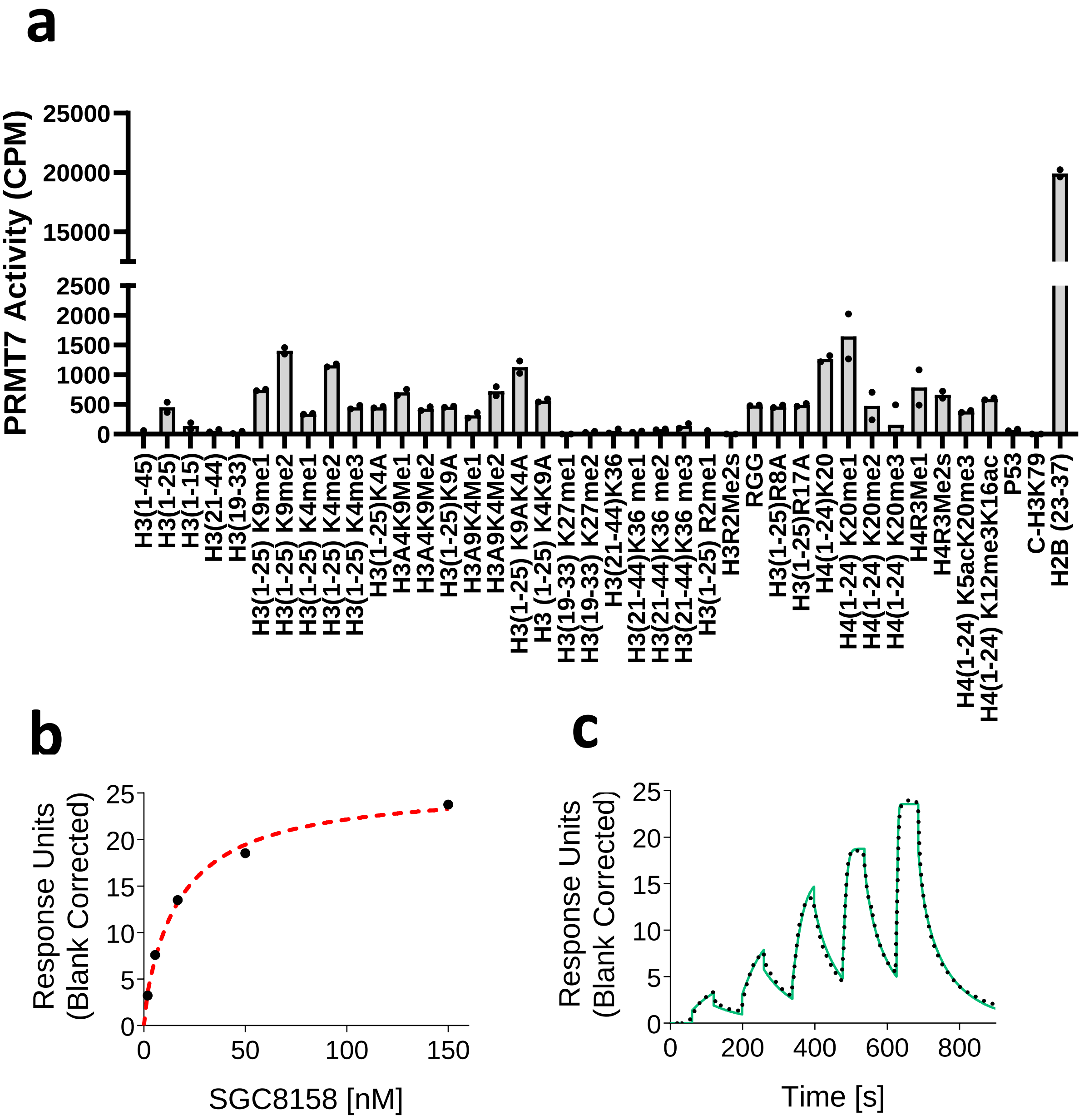

Supplementary Fig. 1: *In vitro* characterization of PRMT7 histone substrate specificity and SGC8158 binding. a, PRMT7 histone substrate specificity. PRMT7 was screened against 28 biotinylated peptides with various lengths and modifications as potential substrates, as described in material and methods. PRMT7 was significantly active only with the H2B (23-37, KKDGKKRKRSRKESY) peptide. Data are presented as average from two independent experiments. **b**,**c** SPR analysis of SGC8158 binding to PRMT7. **b**, The steady state response (black circles) obtained from **c** with the steady state 1:1 binding model fitting (red dashed line). K_D = 16.4 ± 1.0 nM (n=4 biological replicates).**c**, A representative SPR sensorgram (solid green) shown with the kinetic fit (black dots). K_D (6.4 ± 1.2 nM), k_{on} (4.4 ± 1.1 × 10^6 M^{-1} s^{-1}) and k_{off} (2.6 ± 0.5 × 10^{-2} s^{-1}) were calculated from kinetic fitting of 4 biological replicates. Source data are provided as a Source Data file.

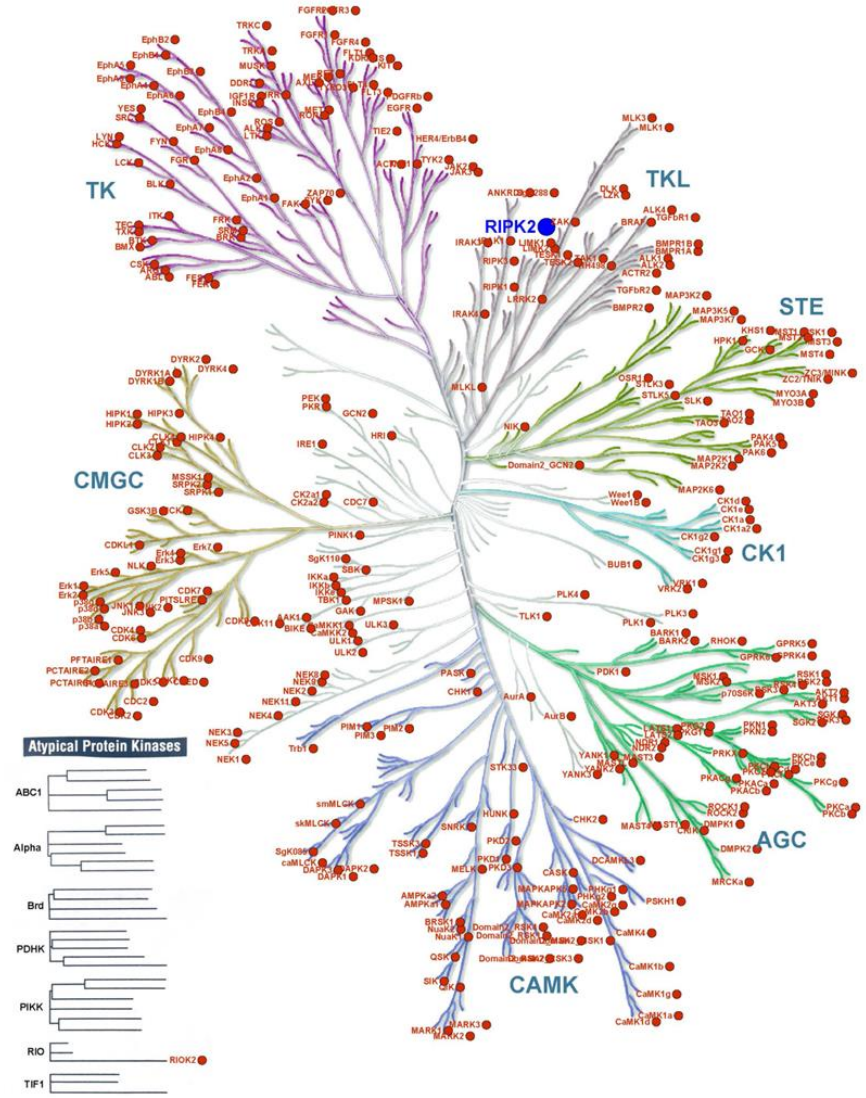

Supplementary Figure 2: Kinase selectivity of SGC8158. Less than 50% inhibition at 1 µM was observed for all kinases except RIPK2: 81% inhibition at 1 µM. Illustration reproduced courtesy of Cell Signaling Technology, Inc. The human protein kinases are from Manning et al [1] Full data can be found in the **Supplementary data 1** relating to this figure.

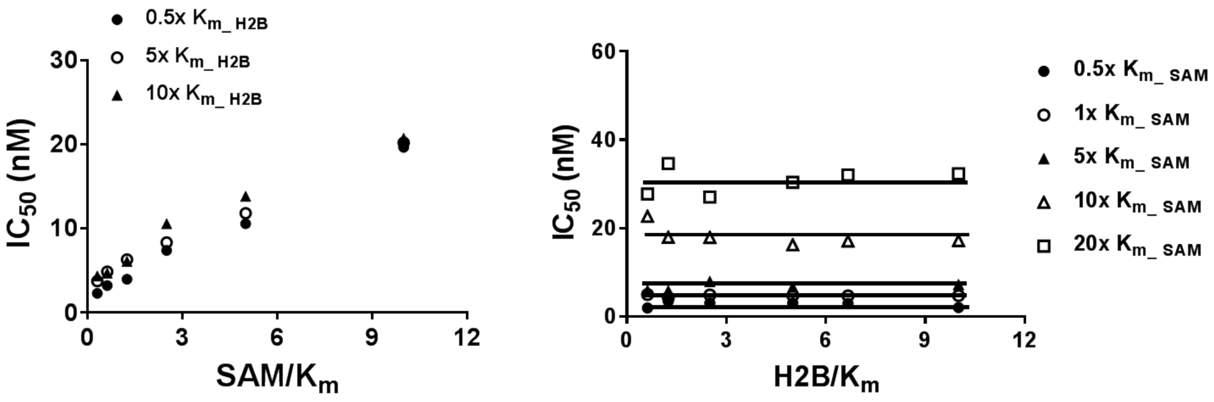

Supplementary Fig. 3: SGC8158 is a SAM competitive PRMT7 inhibitor. SGC8158 MOA determination was performed by extensive titration of substrate (right) and SAM (left) concentrations. IC_{50} values were determined for compound SGC8158 at varying concentrations of SAM, and various fixed concentrations (0.5x, 5x, and 10x K_m) of peptide (K_m for H2B (23-37) is 0.3 ± 0.04 µM); or at varying concentrations of substrate B-H2B and various fixed concentrations (0.5x, 1x, 5x, 10x, and 20x K_m) of SAM (K_m for SAM is 1.1 ± 0.01 µM). The assays were performed using 5 nM PRMT7 (1-692aa), 20 mM Tris-HCl, pH 8.5, 0.01% Tween 20, 5 mM DTT, 2% DMSO, 30 min at 23 ˚C. Source data are provided as a Source Data file.

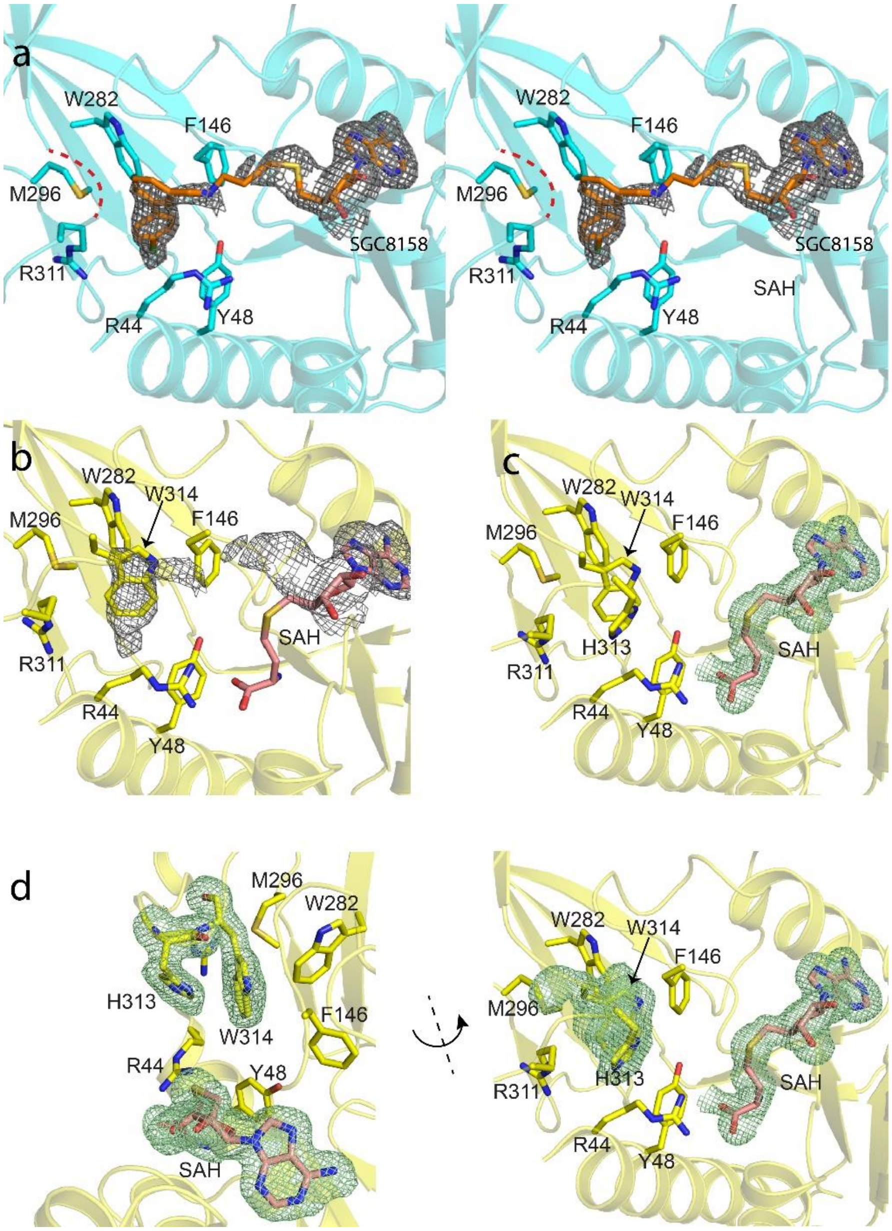

Supplementary Figure 4: Comparison of SGC8158 and SAH omit-maps in *Mm*PRMT7_SGC8158 and *Mm*PRMT7_SAH. a, Stereoview of the SGC8158 binding pocket in *Mm*PRMT7_SGC8158. *Mm*PRMT7 is shown in cartoon representation in Cyan, and SGC8158 in orange. The THW loop is highlighted as red dashed lines representing the unmodeled H313 and W314 residues. The Fo-Fc electron density omit-map for the entire SGC8158 in *Mm*PRMT7_SGC8158 (PDB ID: 6OGN) displayed as a grey mesh, contoured at 2.5σ. **b**, Superposed model of *Mm*PRMT7_SAH (PDB ID: 4C4A) is shown in yellow with SGC8158 omit map same as in **a** (grey mesh). For comparison, SAH is shown as sticks model in pink. **c**, The Fo-Fc electron density omit-map of SAH (PDB ID: 4C4A) displayed in a pale green mesh, contoured at 3.0σ. **d**, The Fo-Fc electron density omit-maps of SAH as well as H313-W314 residues in *Mm*PRMT7_SAH (PDB ID: 4C4A) displayed in pale-green mesh, contoured at 3.0σ. SAH is shown in sticks model in pink.

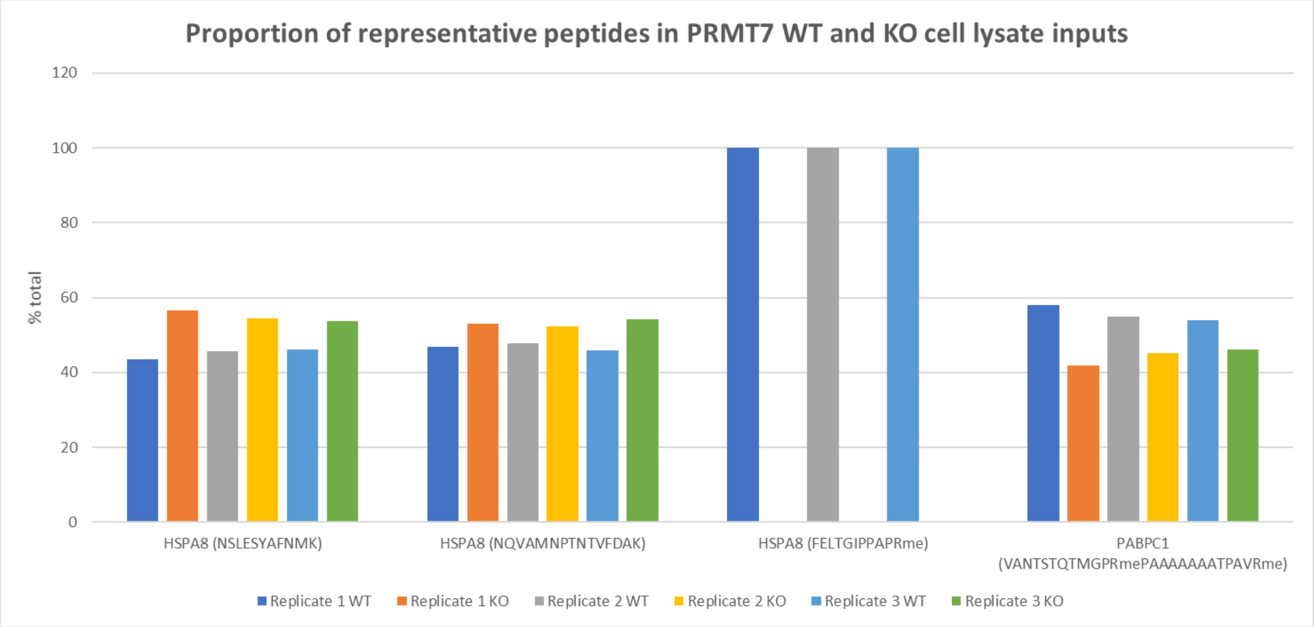

Supplementary Fig. 5 Peptide representation and arginine monomethylation analysis in the input samples. Mass spectrometry analysis of HSPA8 and PABPC1 peptides in PRMT7 wild type (WT) or knockout (KO) cells. HSPA8 proteotypic unmodified peptides NSLESYAFNMK and NQVAMNPTNTVFDAK were used to detect any changes in the total levels of HSPA8. Methylated peptide FELTGIPPAPRme was not detected in the KO samples, while 100% of this peptide was methylated in the WT cells. ND indicates not detected. Approximately half of PABPC1 peptide was methylated and its change due to PRMT7 was not significant. Source data are provided as a Source Data file.

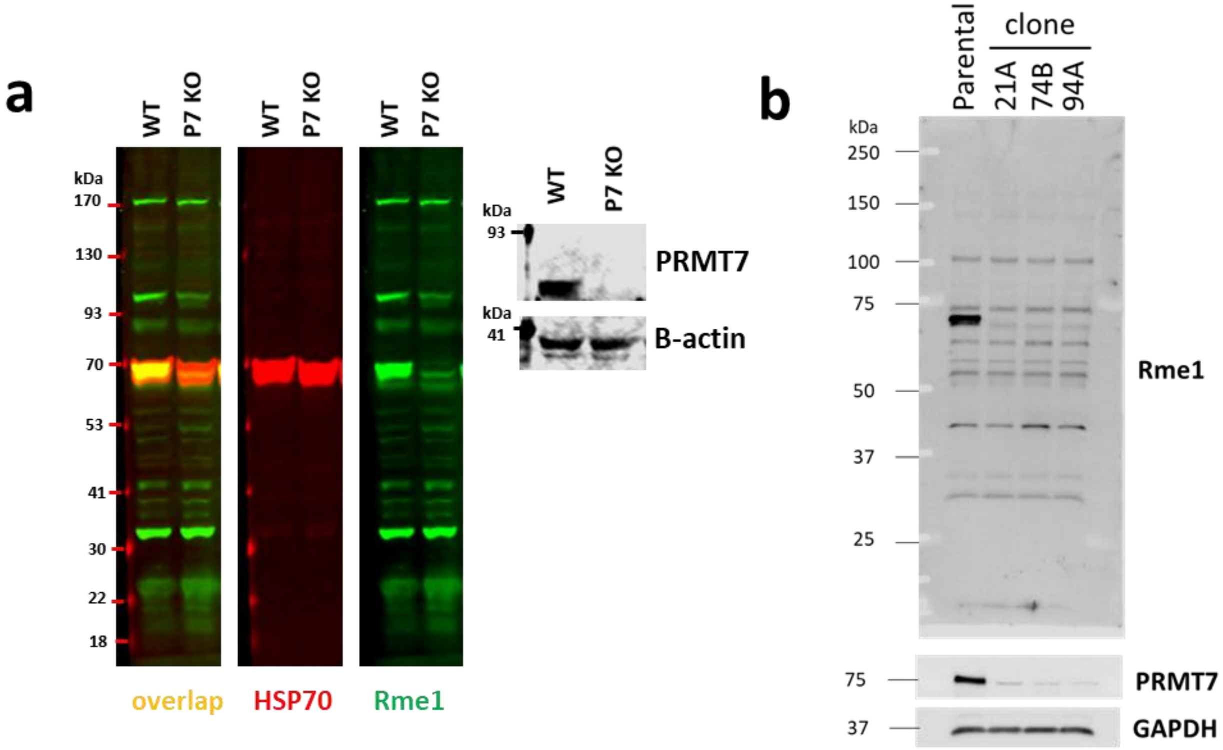

Supplementary Fig. 6: HSP70 family member HSPA8 as potential PRMT7 substrate. a, Western blot analysis of *PRMT7* wild type and knockout HCT116 cell extracts (clone 94A) using monomethyl arginine antibodies (Rme1) shows decrease in 70 kDa band corresponding to HSP70 proteins in *PRMT7* KO cells. **b**, CRISPR clones analysis of *PRMT7* KO in HCT116 cells. Monomethyl arginine signal is diminished at the 70 kDa range. The experiments a and b were repeated independently more than three times with similar results. Source data are provided as a Source Data file.

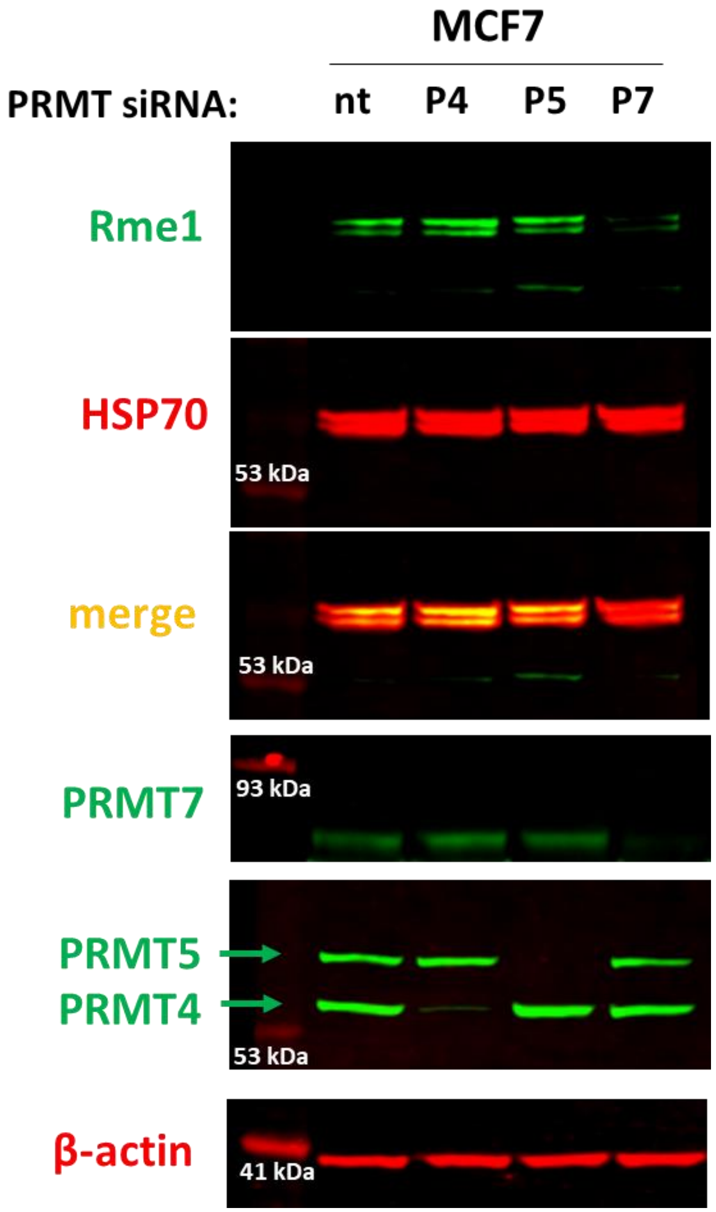

Supplementary Fig. 7: The knockdown of PRMT7 (P7) but not PRMT4 (P4) or PRMT5 (P5) leads to decrease in HSP70 monomethylation in MCF7 cells. MCF7 cells were transfected with siRNA for 3 days and cytoplasmic fraction was analyzed for HSP70 arginine monomethylation (Rme1) levels. **nt**-non-targeting siRNA control. The experiment was repeated independently twice with similar results. Source data are provided as a Source Data file.

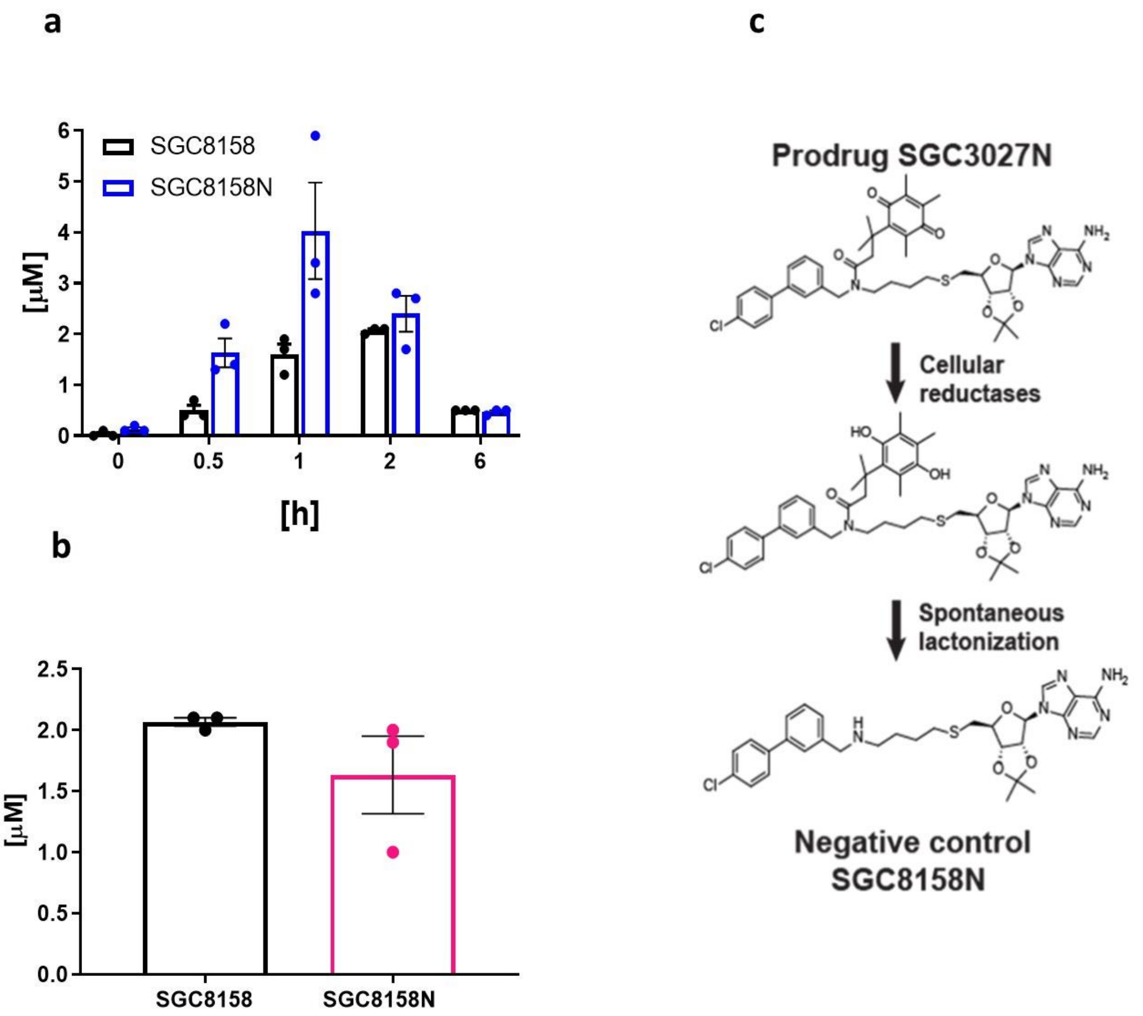

Supplementary. Fig. 8: SGC3027 and SGC3027N are converted in C2C12 cells into active compounds SGC8158 and SGC8158N, respectively. C2C12 cells were treated with 3 µM prodrug compounds for indicated time and the intracellular compound concentration was measured by LC-MS. Relative concentrations are indicated. **a**, The time course of SGC3027 conversion into SGC8158. The results are mean ± SEM of 3 technical replicates. **b**, Cells treated with SGC3027 and SGC3027N for 2 h show similar intracellular concentration of converted compounds SGC8158 and SGC8158N, respectively. The results are mean ± SEM of 3 technical replicates. **c**, Chemical structure and conversion of negative control compound. Source data are provided as a Source Data file.

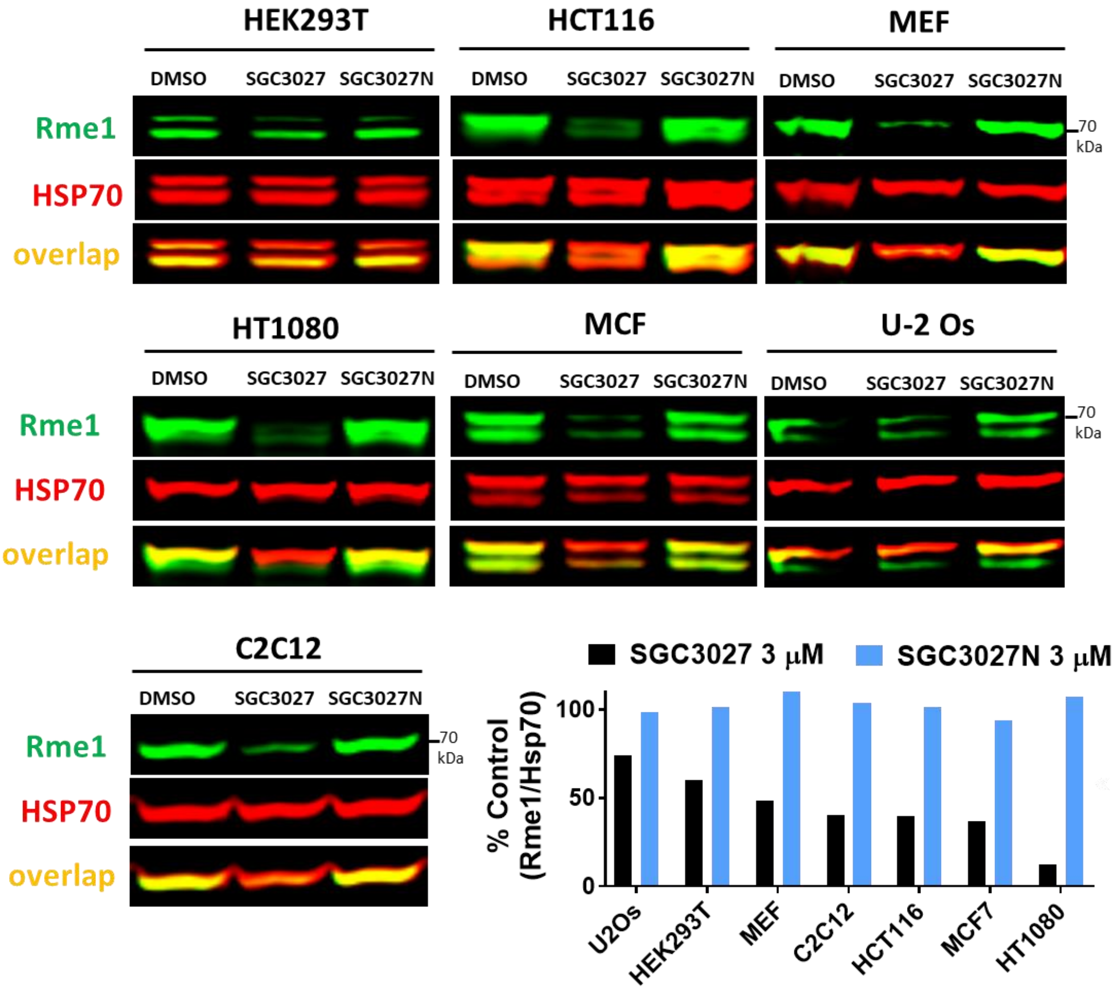

Supplementary Fig. 9: The effect of SGC3027 and SGC3027N on HSP70 monomethylation in different cell lines. Cells were treated with compounds for 2 days and analyzed in western blot for arginine monomethylation (Rme1) and HSP70 levels. SGC3027 cellular potency is cell line dependent. The graph represents ratios of Hsp70-Rme1 band intensities and total HSP70 signal in cells treated with SGC3027 and SGC3027N normalized to untreated control (n=1). Cytoplasmic extracts were used for this experiment as, in some cell lines, the pan-anti-Rme1 antibody used to detect methylation recognized other methylated proteins approximately the same size as Hsp70, thus we recommend using cytoplasmic extracts with longer resolution time. Several cell lines such as HCT116 and MCF7 express the inducible forms HSPA6 and HSPA1 constitutively resulting in 2 bands detected, that both lost methylation upon SGC3027 treatment. Source data are provided as a Source Data file.

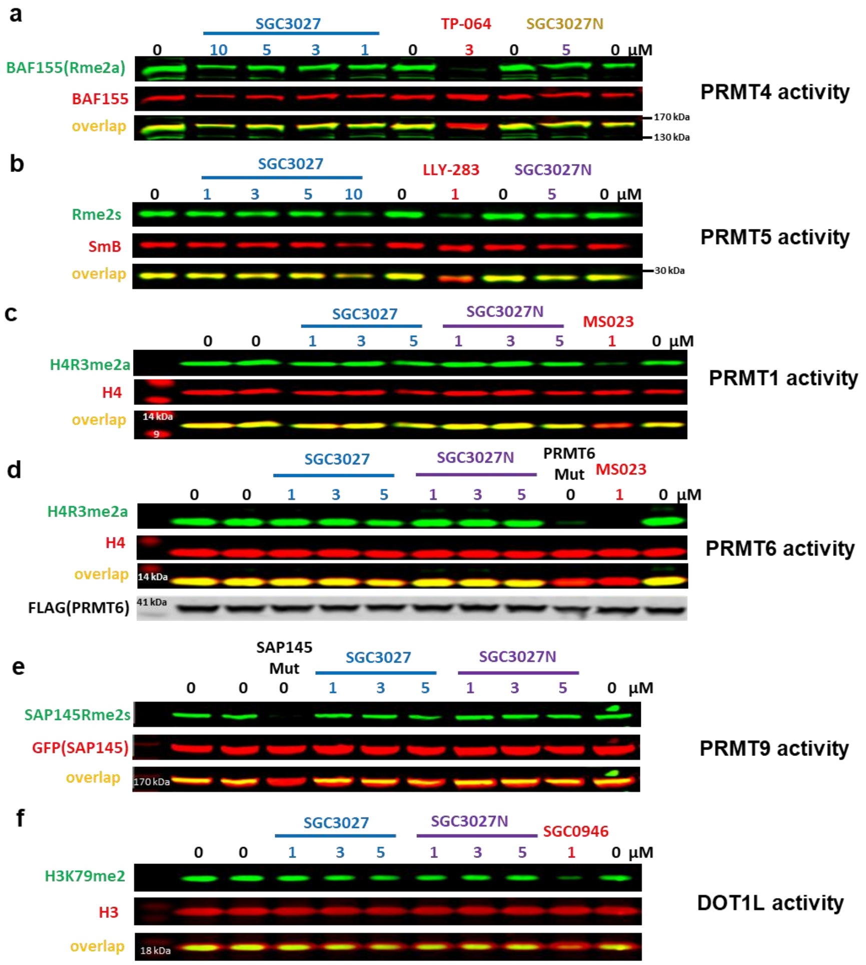

Supplementary Fig. 10: SGC3027 and SGC3027N does not affect PRMT1,4,5,6,9 and DOT1L activity in cells. **a**, PRMT4 dependent BAF155 asymmetric arginine dimethylation levels were only decreased by PRMT4 selective inhibitor TP-064 (C2C12 cells, 2-day treatment). **b**, PRMT5 dependent SmB symmetric arginine dimethylation levels were only decreased by PRMT5 selective inhibitor LLY-283 (C2C12 cells, 2-day treatment). **c**, PRMT1 dependent H4R3 asymmetric arginine dimethylation levels were only decreased by PRMT type I selective inhibitor MS023 (MCF7 cells, 2-day treatment). **d**, PRMT6 dependent H4R3 asymmetric arginine dimethylation levels were only decreased by PRMT type I selective inhibitor MS023. HEK293T cells were transfected with FLAG-tagged PRMT6 WT or Mut(V86K/D88A) and treated with compounds for 20 h. **e**, PRMT9 dependent SAP145 asymmetric arginine dimethylation levels were not affected by compounds treatment. HEK293T cells were transfected with GFP-tagged SAP145 WT or Mut(R508K) and treated with compounds for 20 h**.** **f**, DOT1L dependent H3K79 lysine dimethylation levels were only decreased by DOT1L selective inhibitor SGC0946 (THP-1 cells, 2-day treatment). Experiments a-f were repeated independently twice with similar results. Source data are provided as a Source Data file.

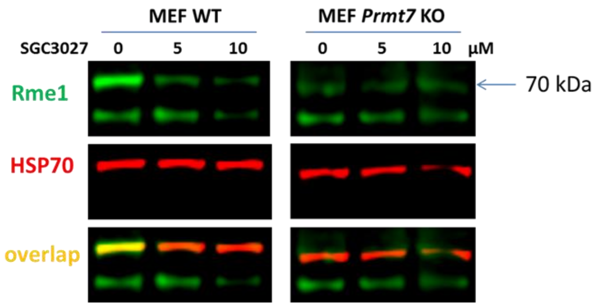

Supplementary Fig. 11: SGC3027 decreases Hsp70 monomethylation in MEF WT cells but not *Prmt7* KO MEFs. Cells were treated with compounds for 2 days. The experiment was repeated independently twice with similar results. Source data are provided as a Source Data file.

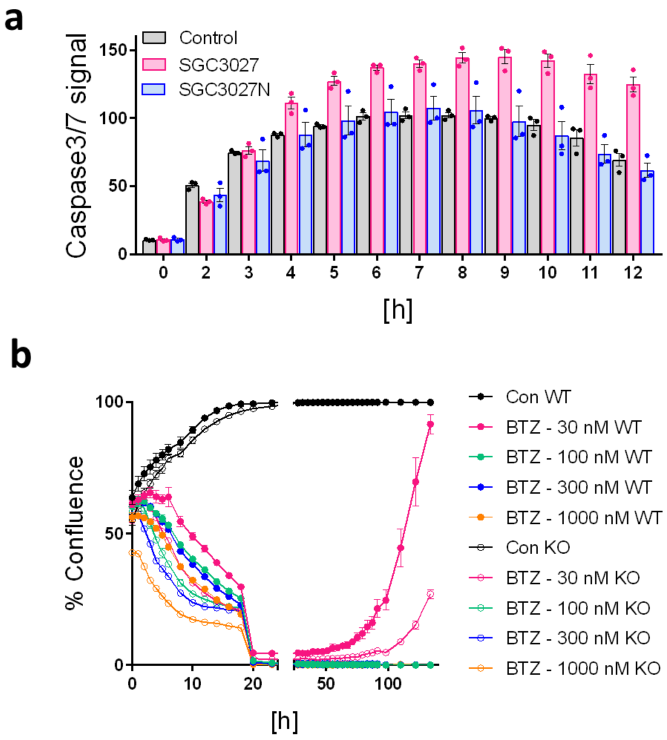

Supplementary Fig. 12: PRMT7 knockout/inhibition affects cell survival after heat shock or proteasomal stress. **a**, SGC3027 inhibition of PRMT7 activity increases apoptosis after heat shock. MEF cells were pretreated with 3 µM compounds for 2 days and heat-shocked for 20 min at 44 °C. Apoptosis was monitored immediately after the heat shock. The results are mean ± SEM of 3 technical replicates **b**, Loss of PRMT7 decreases cell survival after bortezomib (BTZ) treatment for 24h. BTZ was removed after 24 h and the cell confluence was monitored starting 4 h after BTZ treatment. The results are mean ± SEM of 4 (control) and 3(BTZ) technical replicates. Source data are provided as a Source Data file.

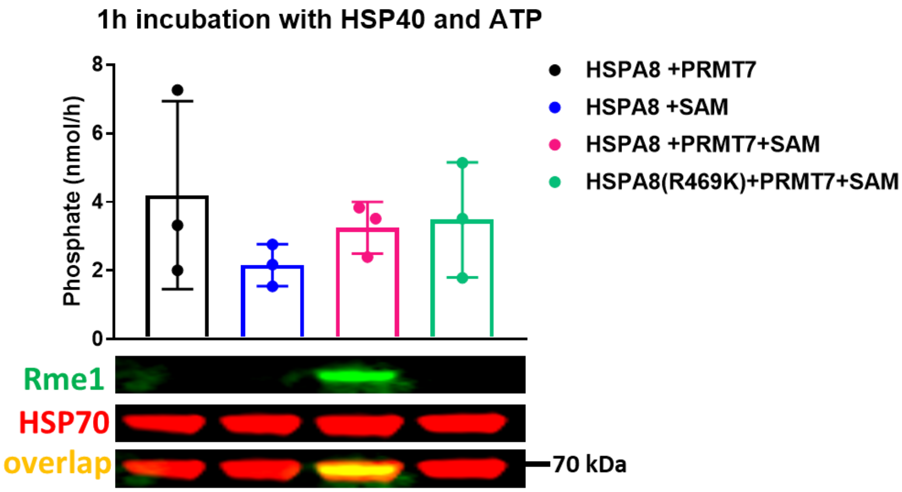

Supplementary Fig. 13: R469 methylation does not influence *in vitro* HSPA8 ATPase activity. Purified HSPA8 and HSPA8 (R469K) proteins were incubated ± PRMT7 and ± SAM for 3 h and subsequently, the ATPase activity was measured using BIOMOL GREEN™ Reagent as well as methylation verified by western blot analysis. The results are mean ± SEM of 3 technical replicates. Source data are provided as a Source Data file.

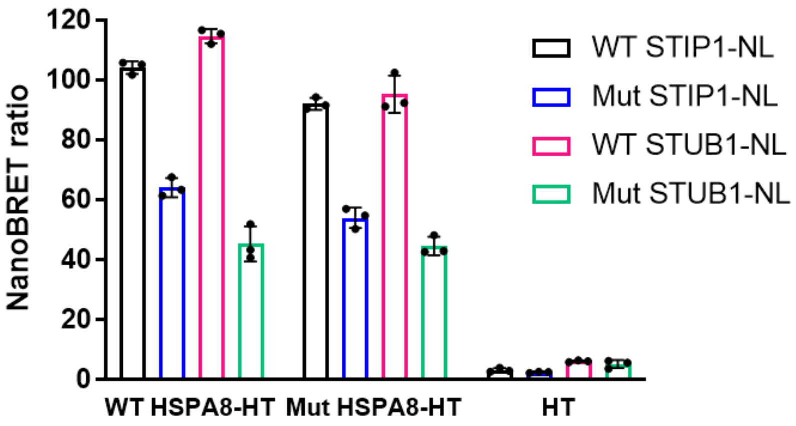

Supplementary Fig. 14: R469 methylation does not influence HSPA8 interaction with STIP1 (HOP) and STUB1 (CHIP) co-chaperones. HEK293T cells were co-transfected with Nanoluc (NL)-tagged STIP1 (WT or K8A mutant) or NL-tagged STUB1 (WT or K30A mutant) and Halo-tagged (HT) HSPA8 (WT or R469K mutant) or HT alone for 24 h. The interaction was measured using NanoBRET assay. The results are mean ± SD of 3 technical replicates. Source data are provided as a Source Data file.

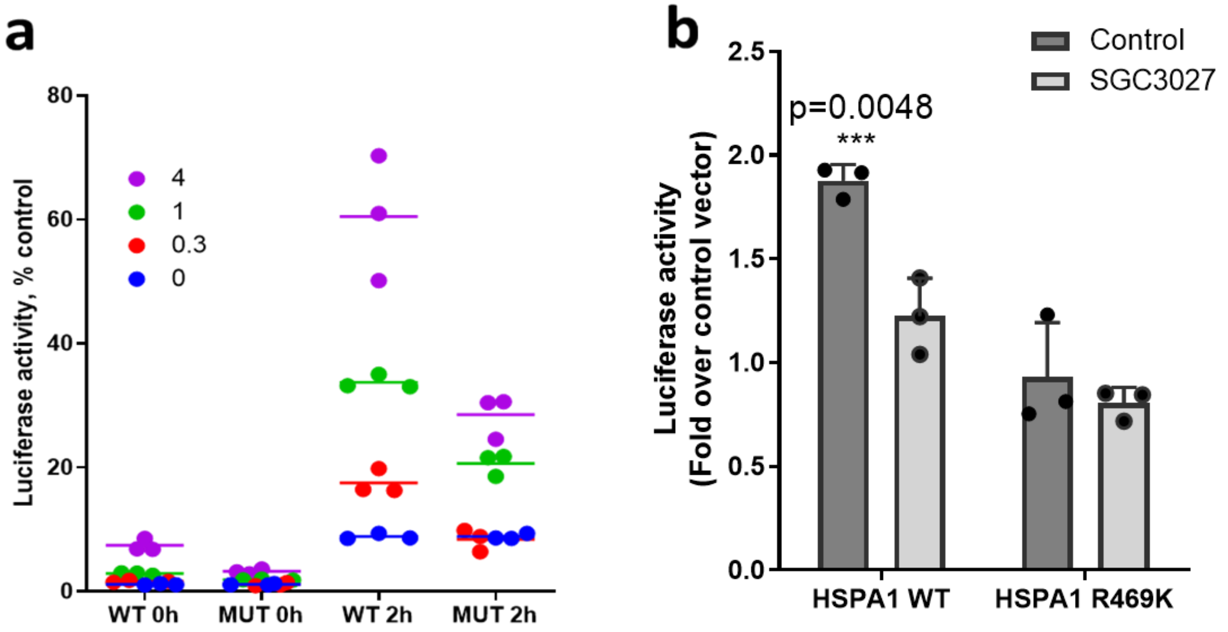

Supplementary Fig. 15: HSPA1 dependent luciferase refolding after heat shock is facilitated by R469 methylation. GFP-HSPA1 WT or R469K mutant were transfected into HEK293T cells and the cells exposed to heat shock. Luciferase activity was measured before and after heat shock with 2 h recovery. **a**, Transfected amounts of HSPA1 WT or R469K (MUT) were titrated (numbers on the legend indicate µg DNA transfected) and luciferase activity assessed at indicated time points. The results are mean of 3 technical replicates **b**, SGC3027 effect on the refolding mediated by the WT or R469K HSPA1. Multiple t-test two-sided analysis was performed with multiple Holm-Sidak comparison correction. The results are mean ± SD of 3 biological replicates. Source data are provided as a Source Data file.

Supplementary Table 1. Selectivity of SGC8172 (n=1). Source data are provided as a Source Data file.* Limit of accurate measurement as protein concentration is 5 nM.

Target	IC_{50} (nM)	Hill Slope
PRMT7	<2.5*	NA
PRMT1	>5000	NA
PRMT3	269	-1.2
PRMT4	3.6	-1
PRMT5-MEP50	44	-0.7
PRMT6	~1200	-1.1
PRMT8	~1500	-1
PRMT9	138	-0.5

Supplementary Table 2. Selectivity of SGC8158 and SGC8158N. Activities of 35 protein, RNA and DNA methyltransferases were assessed in the presence of SGC8158 or SGC8158N at 1 and 10 µM (n=1). The values are presented as percent activity.

Target	Activity %			
	SGC8158		SGC8158N	
	@ 1 µM	@ 10 µM	@ 1 µM	@ 10 µM
**PRMT7**	2	0	95	75
PRMT1	104	67	94	91
PRMT3	57	6	96	96
PRMT4	7	0	101	100
PRMT5	15	2	101	91
PRMT6	100	58	94	87
PRMT8	92	45	97	97
PRMT9	19	4	106	95
G9A	100	99	99	98
GLP	99	95	95	90
SUV39H1	94	90	98	78
SUV39H2	103	102	103	78
SETDB1	103	99	99	91
SETD7	100	100	96	89
MLL1	106	99	101	98
MLL3	105	105	99	100
PRDM9	106	96	101	98
SETD8	101	102	99	100
SUV420H1	96	87	99	102
SUV420H2	98	87	102	101
SETD2	95	86	103	97
PRC2-EZH1	106	100	92	93
PRC2-EZH2	105	94	93	88
SMYD2	101	84	105	104
SMYD3	102	104	100	102
METTL3-14	97	84	87	73
BCDIN3D	55	11	96	94
DNMT1	98	91	94	99
DNMT3A/3L	100	99	100	99
DNMT3B/3L	100	101	104	103
NSD1	104	99	99	98
NSD2	106	101	92	97
NSD3	97	95	100	97
DOT1L	58	13	97	95
ASH1L	96	97	99	102

Supplementary Table 3. Evaluating the selectivity of SGC8158 for hits from Supplementary Table 2. The effect of SGC8158 on activity of PRMTs, BCDIN3D, and DOT1L was assessed by determining the IC_{50} values (n=1). Source data are provided as a Source Data file. * Limit of accurate measurement as protein concentration is 5 nM.

Target	IC_{50} (μM)	Hill Slope
**PRMT7**	<0.0025 *	NA
**PRMT5**	0.15	0.6
**PRMT4**	0.14	1
**PRMT9**	0.16	0.9
**BCDIN3D**	1.1	1
**PRMT3**	1.6	1.4
**DOT1L**	1.7	1
**PRMT8**	7	1
**PRMT6**	15	1.2
**PRMT1**	12	1.2

Supplementary Table 4. Differentially methylated proteins identified by peptide-level mean normalized H/L ratios. The methylated peptides are indicated by the unique protein ID and gene names. Significance was called as H/L ratio of less than -1 (knockout cells (H) relative to control (L)) and the adjusted p-value of less than 0.01. P-values from four independent replicates were calculated by empirical Bayes moderated t-tests and adjusted using the Benjamini-Hochberg procedure as implemented in the Bioconductor package limma (v3.38.3). In the significance column, No indicates no significant difference between WT and KO. The table includes previously reported monomethylated peptides that are also labeled in **Fig 2c**. The four most right columns of the table indicate the corresponding total input protein level changes as the H/L ratio and significance testing. NaN and NA denote levels below detection. The full dataset for the arginine monomethylated peptides can be found in the **Supplementary data 2** and the input protein levels in the **Supplementary data 3**.

Protein	Gene names	logFC	P.Value	adj.P.Val	Rme Site	Significantly Depleted	Phospho SiteDB	Ratio_HL_Repl1	Ratio_HL_Repl2	Ratio_HL_Repl3	Ratio_HL_Repl4	Input_Ratio_HL_Averag	Input P.Value	Input adj.P.Val	Significant Input
Q04637	EIF4G1	-1.3806	9.97E-08	2.15E-05	EIF4G1 (R685)	Yes	Yes	0.383	0.383	0.381	0.389	-0.767	3.69E-07	2.10E-05	No
Q6NUQ4	TMEM214	-2.5946	1.23E-07	2.15E-05	TMEM214 (R29)	Yes	Yes	0.161	0.156	0.174	0.173	NA	NA	NA	NA
P0CG12	CHTF8	-1.3175	3.13E-07	2.74E-05	CHTF8 (R172)	Yes	Yes	0.401	0.407	0.409	0.388	NA	NA	NA	NA
P51610	HCFC1	-1.0272	1.10E-06	6.35E-05	HCFC1 (R524)	Yes	Yes	0.504	0.500	0.484	0.475	0.028	0.733844	0.764197	No
P98179	RBM3	-1.1639	1.24E-06	6.35E-05	RBM3 (R105)	Yes	Yes	0.467	0.431	0.435	0.452	-0.547	1.17E-05	1.48E-04	No
Q04637	EIF4G1	-1.1655	2.04E-06	6.35E-05	EIF4G1 (R1032)	Yes	Yes	0.475	0.436	0.429	0.445	-0.767	3.69E-07	2.10E-05	No
Q2QGD7	ZXDC	-1.8024	2.07E-06	6.35E-05	ZXDC (R13)	Yes	Yes	0.316	0.280	0.290	0.263	NA	NA	NA	NA
P0DMV9/ P17066	HSPA1B;HSP A1A;HSPA6	-7.0941	3.16E-06	7.36E-05	HSPA1B;HSPA1A; HSPA6 (R469)	Yes	Yes	0.006	0.011	0.005	0.009	0.149	4.01E-04	0.001508	No
Q01844	EWSR1	-1.3067	4.06E-06	8.36E-05	EWSR1 (R471)	Yes	Yes	0.394	0.394	0.444	0.387	-0.108	0.005077	0.010848	No
P14678	SNRPB	-1.2421	4.52E-06	8.80E-05	SNRPB (R108)	Yes	Yes	0.425	0.388	0.432	0.449	NA	NA	NA	NA
Q86V81	ALYREF	-2.0604	2.02E-05	2.02E-04	ALYREF (R47)	Yes	Yes	0.238	0.224	0.258	NaN	-0.367	2.04E-05	2.08E-04	No
Q12905	ILF2	-1.1787	2.21E-05	2.15E-04	ILF2 (R24)	Yes	Yes	0.440	NaN	0.429	0.456	-0.246	4.55E-05	3.19E-04	No
Q9BTC0	DIDO1	-1.2664	2.33E-05	2.15E-04	DIDO1 (R1882)	Yes	Yes	0.379	0.432	0.469	0.389	-0.748	4.02E-05	2.94E-04	No
Q9Y3I1	FBXO7	-1.1236	6.37E-05	3.98E-04	FBXO7 (R498)	Yes	Yes	0.477	0.433	0.468	NaN	NA	NA	NA	NA
Q12830	BPTF	-1.2404	5.07E-05	3.35E-04	BPTF (R2155)	Yes	Yes	0.442	0.391	0.490	0.379	NA	NA	NA	NA
Q04637	EIF4G1	-1.4696	1.19E-04	5.69E-04	EIF4G1 (R694)	Yes	Yes	0.358	0.331	NaN	0.398	-0.767	3.69E-07	2.10E-05	No
P11142	HSPA8	-4.3131	3.67E-04	0.001158147	HSPA8 (R469)	Yes	Yes	0.057	0.019	0.096	0.061	0.083	0.004526	0.009871	No
Q9Y520	PRRC2C	-1.1489	0.001964885	0.004298185	PRRC2C (R1186)	Yes	Yes	NaN	0.426	NaN	0.477	NA	NA	NA	NA
Q13435	SF3B2	-1.2550	5.00E-04	0.001458128	SF3B2 (R245)	Yes	Yes	0.451	0.311	0.429	0.512	-0.086	0.021332	0.035095	No
Q96DT7	ZBTB10	-1.4860	0.00119437	0.002882963	ZBTB10 (R126)	Yes	Yes	NaN	0.304	0.333	0.449	NA	NA	NA	NA
Q96QC0	PPP1R10	-1.0510	0.001753912	0.003935059	PPP1R10 (R693)	Yes	Yes	0.410	0.441	0.693	0.433	NA	NA	NA	NA
Q13435	SF3B2	-1.0529	0.002130981	0.004547824	SF3B2 (R247)	Yes	Yes	0.465	0.413	0.700	0.402	-0.086	0.021332	0.035095	No
P07949	RET	-10.1647	2.67E-07	2.74E-05	RET (R878)	Yes	No	0.001	0.001	0.001	0.001	NA	NA	NA	NA
H3BPM6	MKRN2OS	-1.2359	8.46E-05	4.56E-04	MKRN2OS (R73)	Yes	No	0.394	NaN	0.447	0.435	NA	NA	NA	NA
Q5XG87	PAPD7	-1.3063	1.44E-04	6.36E-04	PAPD7 (R125)	Yes	No	NaN	0.367	0.417	0.432	NA	NA	NA	NA
Q9BUT9	FAM195A	-1.2008	1.70E-04	6.68E-04	FAM195A (R65)	Yes	No	0.456	0.405	0.526	0.368	NA	NA	NA	NA
Q8N8M0	NAT16	-8.9513	1.74E-04	6.76E-04	NAT16 (R273)	Yes	No	0.001	0.001	0.001	0.012	NA	NA	NA	NA
Q8N8M0	NAT16	-8.5824	2.29E-04	8.24E-04	NAT16 (R267)	Yes	No	0.007	0.001	0.001	0.008	NA	NA	NA	NA
Q8N8M0	NAT16	-8.6670	3.47E-04	0.001135313	NAT16 (R271)	Yes	No	0.013	0.001	0.001	0.004	NA	NA	NA	NA
Q04637	EIF4G1	-0.9842	1.66E-06	6.35E-05	EIF4G1 (R1042)	No	Yes	0.498	0.529	0.494	0.503	-0.767	3.69E-07	2.10E-05	No
P14678/P63162	SNRPB/SNRPN	-0.8218	2.01E-06	6.35E-05	SNRPB/N (R147)	No	Yes	0.562	0.549	0.575	0.578	-0.033	0.312779	0.367681	No
Q9Y520	PRRC2C	-0.8176	2.18E-06	6.35E-05	PRRC2C (R747)	No	Yes	0.556	0.567	0.560	0.587	NA	NA	NA	NA
Q9UQ35	SRRM2	0.6026	2.94E-06	7.36E-05	SRRM2 (R2342)	No	Yes	1.510	1.525	1.526	1.513	0.044	0.122786	0.16025	No
Q86U42	PABPN1	-0.6874	2.99E-06	7.36E-05	PABPN1 (R17)	No	Yes	0.637	0.617	0.619	0.611	-0.257	2.05E-04	9.51E-04	No
P02545	LMNA	0.9045	3.36E-06	7.36E-05	LMNA (R644)	No	Yes	1.949	1.865	1.788	1.889	-0.029	0.143715	0.184492	No
P22626	HNRNPA2B1	-0.6339	5.31E-06	9.67E-05	HNRNPA2B1 (R26	No	Yes	0.631	0.663	0.646	0.639	NA	NA	NA	NA
Q9UQ35	SRRM2	0.8355	5.53E-06	9.67E-05	SRRM2 (R2221)	No	Yes	1.854	1.715	1.740	1.833	0.044	0.122786	0.16025	No
Q99729	HNRNPAB	-0.5518	6.36E-06	1.06E-04	HNRNPAB (R245)	No	Yes	0.678	0.675	0.697	0.678	-0.496	2.07E-05	2.08E-04	No
P98179	RBM3	-0.5508	7.33E-06	1.13E-04	RBM3 (R131)	No	Yes	0.690	0.673	0.673	0.695	-0.547	1.17E-05	1.48E-04	No
Q9BZZ5	API5	-0.6077	7.58E-06	1.13E-04	API5 (R500)	No	Yes	0.666	0.661	0.664	0.633	-0.057	0.103455	0.137641	No
Q9C0J8	WDR33	-0.5689	8.10E-06	1.13E-04	WDR33 (R1144)	No	Yes	0.692	0.658	0.676	0.671	NA	NA	NA	NA
P22626	HNRNPA2B1	0.9574	8.24E-06	1.13E-04	HNRNPA2B1 (R20	No	Yes	1.942	2.078	1.825	1.931	-0.041	0.063906	0.090654	No
Q92945	KHSRP	-0.7688	8.38E-06	1.13E-04	KHSRP (R411)	No	Yes	0.605	0.599	0.554	0.591	-0.069	0.02098	0.034625	No
Q92945	KHSRP	-0.8446	9.77E-06	1.23E-04	KHSRP (R413)	No	Yes	0.554	0.556	0.592	0.528	-0.069	0.02098	0.034625	No
P22626	HNRNPA2B1	0.4863	1.00E-05	1.23E-04	HNRNPA2B1 (R35	No	Yes	1.386	1.383	1.414	1.420	-0.041	0.063906	0.090654	No
P51991	HNRNPA3	-0.5146	1.04E-05	1.23E-04	HNRNPA3 (R286)	No	Yes	0.693	0.715	0.687	0.705	0.019	0.305385	0.360481	No
Q14103	HNRNPD	-0.5027	1.06E-05	1.23E-04	HNRNPD (R272)	No	Yes	0.710	0.712	0.688	0.713	-0.136	8.10E-04	0.00259	No
Q86V81	ALYREF	-0.7556	1.42E-05	1.57E-04	ALYREF (R197)	No	Yes	0.579	0.562	0.621	0.609	-0.367	2.04E-05	2.08E-04	No
Q9UQ35	SRRM2	0.6927	1.44E-05	1.57E-04	SRRM2 (R2288)	No	Yes	1.652	1.640	1.521	1.656	0.044	0.122786	0.16025	No
Q9BUJ2	HNRNPUL1	-0.4961	1.75E-05	1.80E-04	HNRNPUL1 (R639)	No	Yes	0.734	0.697	0.706	0.700	-0.178	0.006658	0.013458	No
Q14103	HNRNPD	-0.3882	2.29E-05	2.15E-04	HNRNPD (R280)	No	Yes	0.765	0.763	0.754	0.775	-0.136	8.10E-04	0.00259	No
Q92841	DDX17	0.3763	2.54E-05	2.19E-04	DDX17 (R94)	No	Yes	1.289	1.318	1.290	1.295	-0.065	0.020511	0.034054	No
Q99832	CCT7	0.3631	2.57E-05	2.19E-04	CCT7 (R537)	No	Yes	1.279	1.279	1.290	1.297	0.095	0.003193	0.007358	No
Q9NZB2	FAM120A	-0.7255	2.79E-05	2.32E-04	FAM120A (R886)	No	Yes	0.631	0.587	0.569	0.635	-0.238	0.053376	0.077436	No
Q07955	SRSF1	-0.3746	2.85E-05	2.32E-04	SRSF1 (R93)	No	Yes	0.771	0.766	0.764	0.785	0.124	0.001808	0.004757	No
P51991	HNRNPA3	-0.3701	3.05E-05	2.43E-04	HNRNPA3 (R214)	No	Yes	0.767	0.771	0.769	0.789	0.019	0.305385	0.360481	No
Q1KMD3	HNRNPUL2	0.6082	3.23E-05	2.51E-04	HNRNPUL2 (R747)	No	Yes	1.614	1.464	1.545	1.479	-0.150	0.001964	0.00507	No
O43809	NUDT21	0.3905	3.64E-05	2.74E-04	NUDT21 (R15)	No	Yes	1.349	1.297	1.303	1.294	-0.007	0.685021	0.722816	No
Q14103	HNRNPD	-0.4791	3.68E-05	2.74E-04	HNRNPD (R278)	No	Yes	0.684	0.732	0.730	0.724	-0.136	8.10E-04	0.00259	No
O95429	BAG4	-0.6147	3.94E-05	2.87E-04	BAG4 (R53)	No	Yes	0.681	0.678	0.639	0.616	NA	NA	NA	NA

Supplementary Table 5. Characterization of PRMT7 catalytic mutant clone. Clone 32 was genotyped by Sanger sequencing PCR-amplified TA-cloned genomic DNA. We have found three mutations in clone 32 genome which is consistent with near tetraploid karyotype of C2C12[1].

	Exon3 mutation	Exon3 and 4 amino acid sequence	Type of mutation
PRMT7		SSYADMLHDKDRNIKYYQGIRAAVSRVKDRGQKALVLDIGTGTG LLSMMAVTAGADFCYAIE	
32-1	DelCCGAC	SSYDAT-QRQKYKILPGYPGSCEQGERQRTEGLGS- HWHWHRPLVNDGSYCRG-LLLCYR	premature stop codon in exon 3
32-2	DelATG	SSSDMLHDKDRNIKYYQGIRAAVSRVKDRGQKALVLDIGTGTGLL SMMAVTAGADFCYAIE	Y35del and A36S mutation
32-3	DelCCGAC	SSYDAT-QRQKYKILPGYPGSCEQGERQRTEGLGS- HWHWHRPLVNDGSYCRG-LLLCYR	premature stop codon in exon 3
32-4	DelCCGAC	SSYDAT-QRQKYKILPGYPGSCEQGERQRTEGLGS- HWHWHRPLVNDGSYCRG-LLLCYR	premature stop codon in exon 3
32-5	DelTATG	SSPTCYMTKTEI-NTTRVSGQL-AG- KTEDRRPWFLTLALAQASCQ-WQLLQGLTSAMLS	premature stop codon in exon 4
32-6	DelTATG	SSPTCYMTKTEI-NTTRVSGQL-AG- KTEDRRPWFLTLALAQASCQ-WQLLQGLTSAMLS	premature stop codon in exon 4
32-7	DelATG	SSSDMLHDKDRNIKYYQGIRAAVSRVKDRGQKALVLDIGTGTGLL SMMAVTAGADFCYAIE	Y35del and A36S mutation
32-8	Del ATG	SSSDMLHDKDRNIKYYQGIRAAVSRVKDRGQKALVLDIGTGTGLL SMMAVTAGADFCYAIE	Y35del and A36S mutation
32-9	DelTATG	SSPTCYMTKTEI-NTTRVSGQL-AG- KTEDRRPWFLTLALAQASCQ-WQLLQGLTSAMLS	premature stop codon in exon 4

Supplementary Table 6. Data collection and refinement statistics.

	*Mm*PRMT7_SGC8158 (PDB ID:6OGN)
**Data collection**	
Space group	$P4_32_12$
Cell dimensions	
a, *b*, *c* (Å)	97.8, 97.8, 169.5
α, β, γ (°)	90.0, 90.0, 90.0
Resolution (Å)	47.03 - 2.40 (2.44-2.40)*
R_{sym} or R_{merge}	0.114 (1.172)
$I / \sigma I$	32.8 (1.85)
$CC_{1/2}$	0.99 (0.68)
Completeness (%)	99.7 (100.0)
Redundancy	12.9 (12.2)
**Refinement**	
Resolution (Å)	47.03 - 2.40
No. reflections	31243
R_{work} / R_{free}	20.8/26.1
No. atoms	
Protein	4705
Ligand/ion	38
Water	42
B-factors	
Protein	70.9
Ligand/ion	71.3
Water	56.1
R.m.s. deviations	
Bond lengths (Å)	0.009
Bond angles (°)	1.365

*Values in parentheses are for highest-resolution shell.

Supplementary Table 7. A typical LC-MS and LC-MSMS setting for quantitative acquisition. Q-TOF LC-MS and MSMS Conditions.

Detector:	Waters SYNAPT G2-S (UEB202)
Polarity:	ES+
Resolution:	20000
Capillary (kV):	2.0000
Source Temperature (°C):	150
Sampling Cone:	20.0000
Source Offset:	70.0000
Desolvation Temperature (°C):	500
Cone Gas Flow (L/Hr):	150.0
Desolvation Gas Flow (L/Hr):	650.0
Nebuliser Gas Flow (Bar):	6.0
Trap Collision Energy:	4.0
Acquisition mass range:	
Start mass	100.000
End mass	1200.000
Calibration mass range:	
Start mass	72.134
End mass	1285.550
Function Parameters:	Function 1 - TOF MS FUNCTION
Scan Time (sec):	0.300
Interscan Time (sec):	0.015
Start Mass:	100.0
End Mass:	1200.0
Start Time (mins)	0.00
End Time (mins)	6.50
Data Format	Continuum
Analyser	Resolution Mode
Function Parameters: -	Function 2 - TOF MSMS FUNCTION
Set Mass:	586.13
Product Ion:	569.14
ADC Sample Frequency (GHz):	3.0
ADC Pusher Frequency (µs):	54.0
ADC Pusher Width (µs):	1.50
Use Tune Page Cone Voltage:	YES
Trap Collision Energy (eV):	30.0
Transfer Collision Energy (eV):	5.0
Sensitivity:	Normal
Dynamic Range:	Normal
Fragmentation Mode:	CID

Supplementary Methods

PRMT7 chemical probe synthesis: Experimental Procedures and Characterization data

General Considerations. Reactions were carried out under nitrogen or argon atmosphere with dry solvents using anhydrous conditions unless otherwise stated. All solvents were purchased from commercial sources and used as received. All fine chemicals were obtained from Sigma-Aldrich or Combi-Blocks and used without further purification unless otherwise stated. Yields refer to chromatographically and spectroscopically (^{1}H-NMR) homogeneous materials unless otherwise stated. NMR spectra were recorded on a Bruker AV-500 spectrometer and calibrated using residual undeuterated solvent as an internal reference ($CDCl_3$ @ δ 7.26 ppm ^{1}H NMR, CD_3OD @ δ 3.31 ppm ^{1}H NMR).

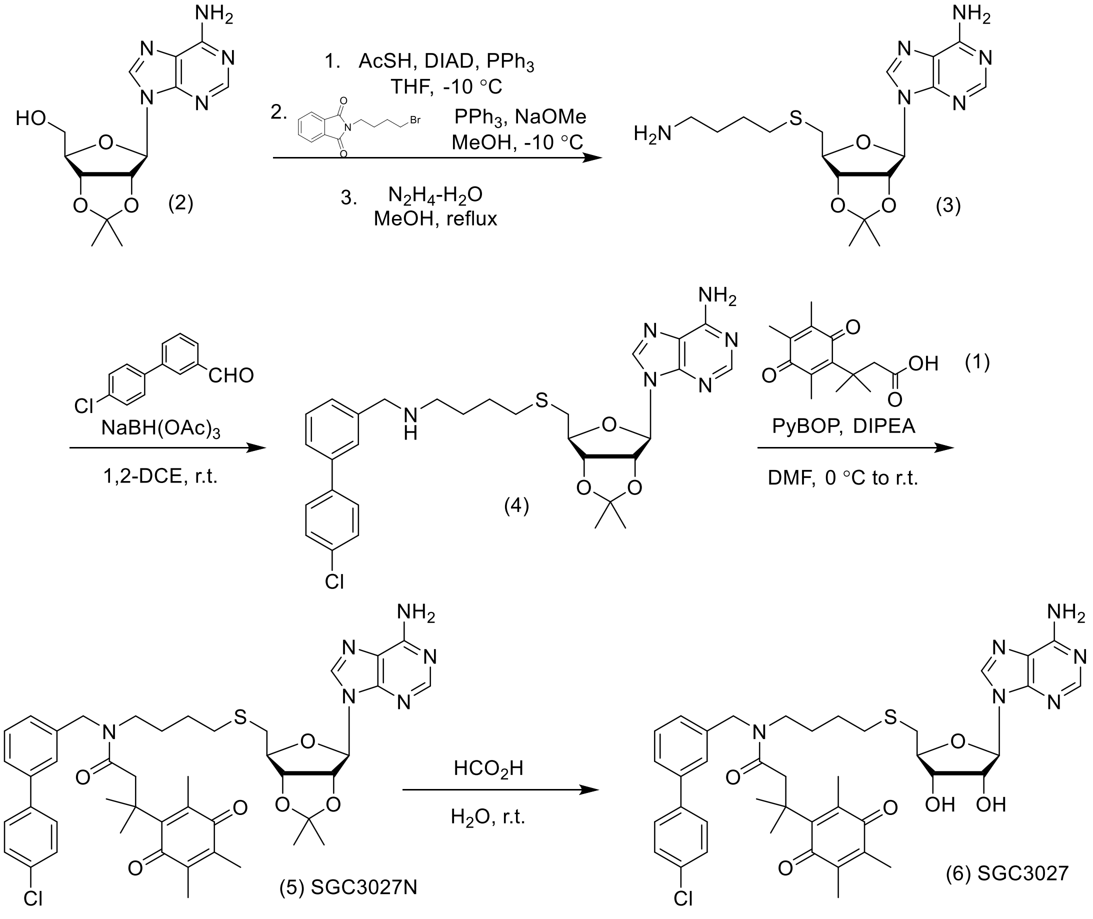

Supplementary Figure 16. Synthesis of the PRMT7 chemical probe.

6-Hydroxy-4,4,5,7,8-pentamethylchroman-2-one

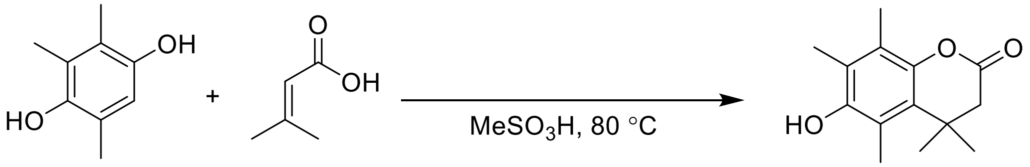

A solution of 3,3-dimethylacrylic acid (1.97 g, 19.7 mmol) and trimethylhydroquinone (3.00 g, 19.7 mmol) in methanesulfonic acid (5 mL) was heated at 70 °C in an oil bath with stirring for 2 h. The mixture was poured into water (100 mL) and extracted with EtOAc (3 x 25 mL). The combined extracts were washed with water (50 mL), saturated $NaHCO_3$ solution (2 x 50 mL), and saturated NaCl solution (50 mL) then dried over Na_2SO_4 prior to removal of solvent under reduced pressure to give 6-hydroxy-4,4,5,7,8-pentamethylchroman-2-one (4.47 g, 97 % yield) as a colorless crystalline solid. ^1H NMR (500 MHz, $CDCl_3$) δ 4.61 (d, *J* = 2.4 Hz, 1H), 2.55 (s, 2H), 2.36 (s, 3H), 2.22 (s, 3H), 2.18 (s, 3H), 1.45 (s, 6H); LCMS RT: 1.81 min; MS (*m/z*): $[MH]^+$ calcd. for $C_{14}H_{19}O_3$, 235.1; found, 235.2; Purity (254 nm): 99%.

3-Methyl-3-(2,4,5-trimethyl-3,6-dioxocyclohexa-1,4-dien-1-yl)butanoic acid (1)

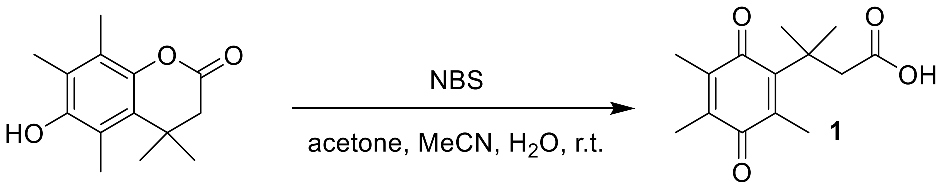

To a solution of 6-hydroxy-4,4,5,7,8-pentamethylchroman-2-one (0.501 g, 2.13 mmol) dissolved in a mixture of acetonitrile (18 mL), acetone (4 mL), and water (18 mL) was added *N*-bromosuccinimide (0.418 g, 2.347 mmol) in three portions, and the mixture was stirred at room temperature for 30 min. After removal of the organic solvents under a stream of nitrogen overnight, the remaining yellow crystalline solid suspended in water was collected by vacuum filtration and washed with water (2 x 20 mL) to give **1** (0.495 g, 93 % yield) as a yellow crystalline solid. ^1H NMR (500 MHz, $CDCl_3$) δ 3.02 (s, 2H), 2.14 (s, 3H), 1.95 (s, 3H), 1.93 (s, 3H), 1.43 (s, 6H); LCMS RT: 1.75 min MS (*m/z*): $[MH]^+$ calcd. for $C_{14}H_{19}O_4$, 251.1; found, 251.5; Purity (254 nm): 99%.

9-((3a*R*,4*R*,6*S*,6a*S*)-6-(((4-Aminobutyl)thio)methyl)-2,2-dimethyltetrahydrofuro[3,4-*d*][1,3]dioxol-4-yl)-9*H*-purin-6-amine (3)

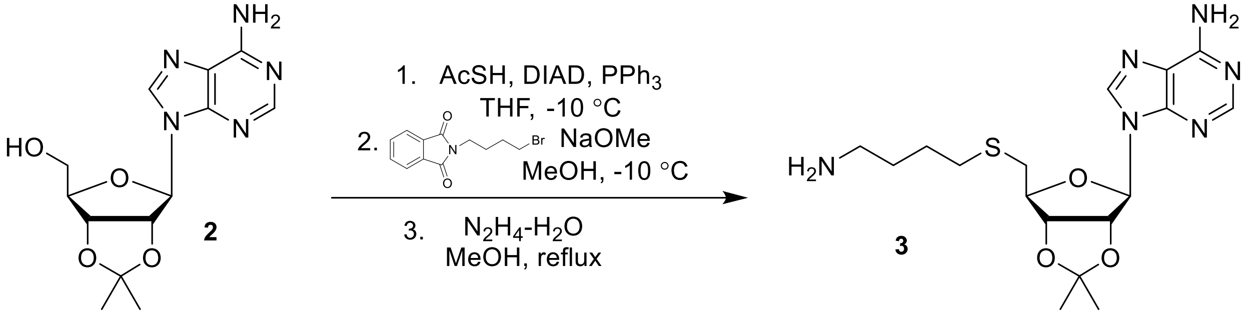

To a solution of triphenylphosphine (9.39 g, 35.8 mmol) in THF at –10 °C was added diisopropyl azodicarboxylate (7.05 mL, 35.8 mmol) over 15 minutes, and the resulting slurry was stirred vigorously. After 20 minutes at this temperature, 2',3'-*O*-isopropylideneadenosine (5.00 g, 16.3 mmol) was added as a solid in one portion, and the reaction mixture was stirred for an hour prior to addition of thioacetic acid (2.56 mL, 35.8 mmol). The reaction mixture was allowed to stir for another hour prior to concentration under reduced pressure. The crude product was then column chromatographed (silica gel, CH_2Cl_2/MeOH, 100:0 to 95:5 v/v) to afford the intermediate thioester (4.88 g, 82% yield) as a colorless oil. The crude product contained triphenylphosphine oxide was used in subsequent reactions without further purification. LCMS RT: 1.39 min; MS (*m/z*): $[MH]^+$ calcd. for $C_{15}H_{20}N_5O_4S$, 366.1; found, 365.9; Purity (254 nm): >95%.

To the thioester (4.02 g, 11.0 mmol) produced above in MeOH (40 mL) were added *N*-(4-bromobutyl)phthalimide (4.04 g, 14.3 mmol), triphenylphosphine (0.290 g, 1.10 mmol), and DMF (5 mL) prior to cooling the solution to –10 °C. After stirring at this temperature for 15 minutes, NaOMe (30% w/v in MeOH, 4.08 mL, 22.0 mmol) was then added. The reaction was then allowed to warm to room temperature over 1 h and stirred at this temperature for 5 h, at which point alkylation was complete and no starting material remained. The reaction mixture was then further diluted with MeOH (50 mL). To the mixture was added hydrazine monohydrate (5.34 mL, 110 mmol), and the solution was heated to reflux for 30 minutes, at which point LCMS showed complete deprotection of the phthalimide protecting group. Upon cooling to room temperature, Celite was added, and all volatiles removed under reduced pressure. The dry-loaded product was then purified by column chromatography (RP-C18, H_2O (0.5% NH_4OH)/MeCN, 98:2 to 10:90 v/v) to afford **3** (2.91 g, 67% yield) as a colorless powder. ^1H NMR (500 MHz, CD_3OD) δ 8.30 (s, 1H), 8.25 (s, 1H), 6.20 (d, *J* = 2.0 Hz, 1H), 5.57 (dd, *J* = 6.3, 2.1 Hz, 1H), 5.08 (dd, *J* = 6.2, 2.8 Hz, 1H), 4.36 (td, *J* = 6.8, 2.8 Hz, 1H), 2.80 (d, *J* = 6.9 Hz, 2H), 2.57 (t, *J* = 6.7 Hz, 2H), 2.49 (t, *J* = 6.8 Hz, 2H), 1.60 (s, 3H), 1.55 – 1.42 (m, 4H), 1.41 (s, 3H); LCMS RT: 1.26 min; MS (*m/z*): $[MH]^+$ calcd. for $C_{17}H_{27}N_6O_3S$, 395.2; found, 395.5; Purity (254 nm): >95%.

9-((3a*R*,4*R*,6*S*,6a*S*)-6-(((4-(((4'-Chloro-[1,1'-biphenyl]-3-yl)methyl)amino)butyl)thio)methyl)-2,2-dimethyltetrahydrofuro[3,4-*d*][1,3]dioxol-4-yl)-9*H*-purin-6-amine (4)

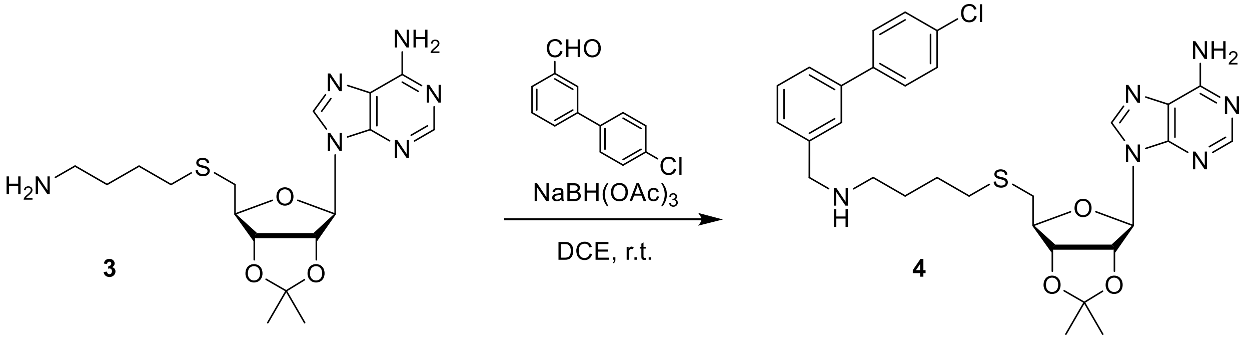

To a solution of **3** (115 mg, 0.292 mmol) and 4'-chloro-[1,1'-biphenyl]-3-carbaldehyde (63.2 mg, 0.292 mmol) in 1,2-dichloroethane (10 mL) was added sodium triacetoxyborohydride (93.0 mg, 0.437 mmol) in one portion at room temperature. The mixture was stirred for 2 h prior to addition of a saturated solution of aqueous $NaHCO_3$ (20 mL). The mixture was extracted with EtOAc (3 x 10 mL), and the combined organic layers dried over Na_2SO_4 prior to concentration under reduced pressure to afford a crude oil which was column chromatographed (silica gel, CH_2Cl_2/MeOH/NH_4OH, 100:0:0 to 96.7:3:0.3 v/v) to give **4** (0.142 g, 82 % yield) as a colorless semi-solid. ^1H NMR (500 MHz, $CDCl_3$) δ 8.35 (s, 1H), 7.94 (s, 1H), 7.56 (s, 1H), 7.53 (d, *J* = 8.5 Hz, 2H), 7.45 (d, *J* = 7.6 Hz, 1H), 7.42 – 7.37 (m, 3H), 7.33 (d, *J* = 7.3 Hz, 1H), 6.07 (d, *J* = 2.1 Hz, 1H), 5.62 (s, 2H), 5.52 (dd, *J* = 6.4, 2.1 Hz, 1H), 5.04 (dd, *J* = 6.4, 3.0 Hz, 1H), 4.38 (td, *J* = 6.8, 3.0 Hz, 1H), 3.87 (s, 2H), 2.80 (dd, *J* = 13.6, 7.1 Hz, 1H), 2.74 (dd, *J* = 13.6, 6.6 Hz, 1H), 2.65 (t, *J* = 7.0 Hz, 2H), 2.47 (t, *J* = 6.6 Hz, 2H), 1.64 – 1.51 (m, 7H), 1.39 (s, 3H); LCMS RT: 1.58 min; MS (m/z): $[MH]^+$ calcd. for $C_{30}H_{36}ClN_6O_3S$, 595.2; found, 595.7; Purity (254 nm): 99%.

N-(4-((((3aS,4S,6R,6aR)-6-(6-amino-9H-purin-9-yl)-2,2-Dimethyltetrahydrofuro[3,4-d][1,3]dioxol-4-yl)methyl)thio)butyl)-N-((4'-chloro-[1,1'-biphenyl]-3-yl)methyl)-3-methyl-3-(2,4,5-trimethyl-3,6-dioxocyclohexa-1,4-dien-1-yl)butanamide (5) (SGC3027N)

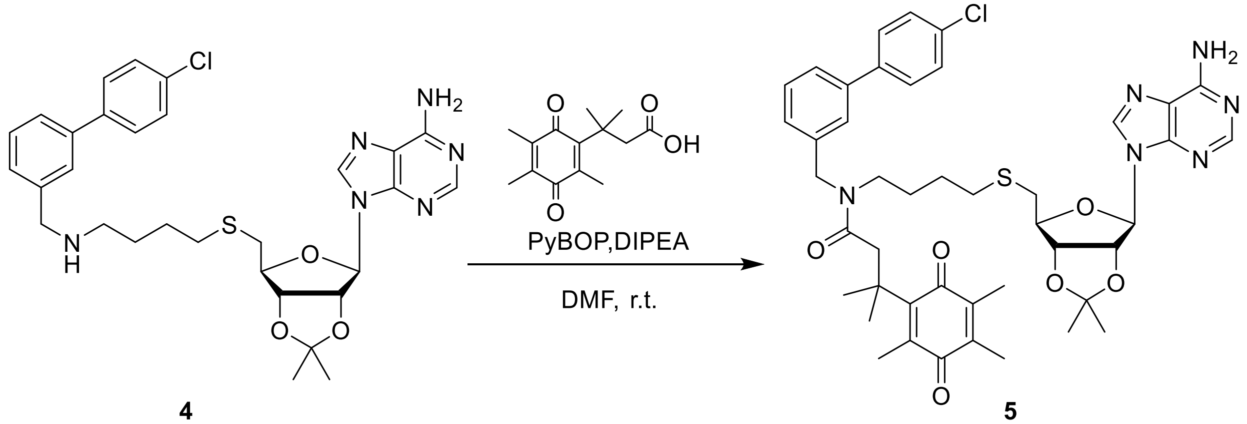

4 **5**

To a solution of **4** (62.0 mg, 0.104 mmol) in *N*,*N*-dimethylformamide (DMF) (1 mL) were added 3-methyl-3-(2,4,5-trimethyl-3,6-dioxocyclohexa-1,4-dien-1-yl)butanoic acid (26.1 mg, 0.104 mmol) and triethylamine (36 µL, 0.260 mmol). The mixture was cooled to 0 °C prior to addition of PyBOP (81 mg, 0.156 mmol), and the mixture was stirred for 3 h at this temperature. The reaction was quenched with a saturated solution of brine (20 mL), and the aqueous phase was extracted with EtOAc (3 x 10 mL). The combined organic layers were dried over Na_2SO_4, concentrated under reduced pressure, and the yellow residue was column chromatographed (silica gel, CH_2Cl_2/MeOH/NH_4Cl, 100:0:0 to 95.6:4:0.4 v/v) to afford SGC3027N **(5)** (66 mg, 77 % yield) as a bright yellow oil (mixture of isomers). ^{1}H NMR (500 MHz, $CDCl_3$) δ 8.33 (d, *J* = 4.2 Hz, 1H), 7.91 (d, *J* = 1.8 Hz, 1H), 7.59 (d, *J* = 8.5 Hz, 1H), 7.52 – 7.44 (m, 2H), 7.44 – 7.38 (m, 3H), 7.38 – 7.31 (m, 1H), 7.22 – 7.09 (m, 1H), 6.07 (dd, *J* = 7.8, 2.0 Hz, 1H), 5.99 (d, *J* = 12.1 Hz, 2H), 5.52 (ddd, *J* = 15.4, 6.4, 2.0 Hz, 1H), 5.05 (ddd, *J* = 19.8, 6.4, 3.1 Hz, 1H), 4.55 (d, *J* = 11.9 Hz, 2H), 4.42 – 4.31 (m, 1H), 3.25 – 3.16 (m, 2H), 3.09 (d, *J* = 17.2 Hz, 2H), 2.87 – 2.67 (m, 2H), 2.54 – 2.44 (m, 2H), 2.17 – 2.10 (m, 3H), 1.95 – 1.75 (m, 6H), 1.67 – 1.60 (m, 4H), 1.56 – 1.49 (m, 2H), 1.48 – 1.42 (m, 4H), 1.41 – 1.37 (m, 6H); ^{13}C NMR (126 MHz, $CDCl_3$) δ 191.43, 187.84, 172.37, 155.67, 154.87, 153.31, 149.43, 143.32, 140.93, 140.21, 139.49, 138.48, 137.57, 136.11, 133.89, 129.62, 129.13, 128.59, 126.34, 125.74, 124.88, 120.50, 114.58, 91.06, 86.94, 84.16, 83.96, 51.07, 46.42, 37.65, 34.49, 32.44, 28.68, 27.24, 26.74, 26.59, 25.48, 14.30, 12.60, 12.25. Complicated by rotamers (major rotamer reported); LCMS RT: 2.59 min; MS (*m/z*): $[MH]^+$ calcd. for $C_{44}H_{52}ClN_6O_6S$, 827.3; found, 828.0; Purity (254 nm): 99%.

***N*-(4-((((2*S*,3*S*,4*R*,5*R*)-5-(6-Amino-9*H*-purin-9-yl)-3,4-dihydroxytetrahydrofuran-2-yl)methyl)thio)butyl)-*N*-((4'-chloro-[1,1'-biphenyl]-3-yl)methyl)-3-methyl-3-(2,4,5-trimethyl-3,6-dioxocyclohexa-1,4-dien-1-yl)butanamide (6) (SGC3027)**

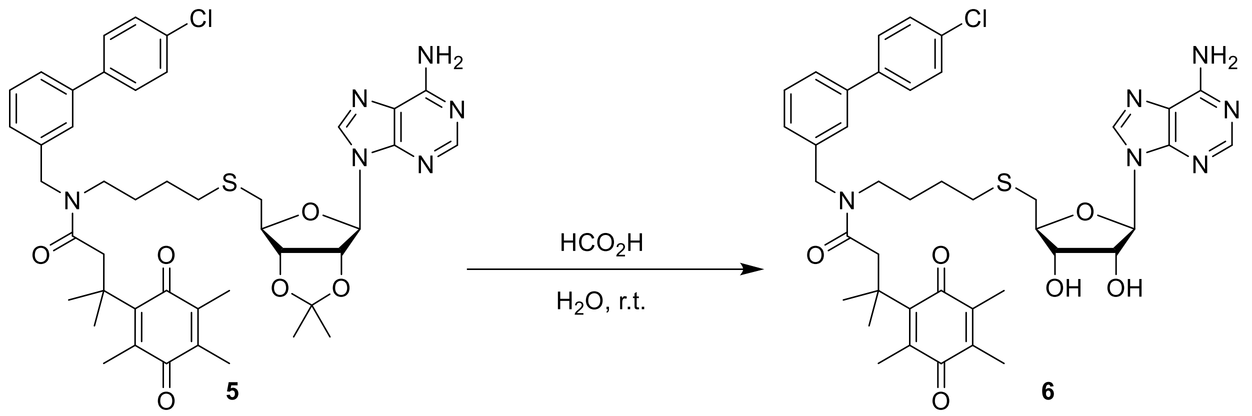

A solution of **5** (25.7 mg, 0.031 mmol) in HCO_2H/H_2O (2 mL, 4:1 v/v) was stirred overnight at room temperature. All volatiles were then evaporated, and the crude yellow residue column chromatographed (silica gel, CH_2Cl_2/MeOH/NH_4OH, 100:0:0 to 89:10:1 v/v) to afford SGC3027 **(6)** (21 mg, 86 % yield) as a bright yellow oil, which solidified on standing (mixture of rotamers). ^{1}H NMR (500 MHz, $CDCl_3$) δ 8.18 (s, 1H), 8.00 (d, *J* = 1.9 Hz, 1H), 7.57 (d, *J* = 8.5 Hz, 1H), 7.51 – 7.42 (m, 2H), 7.41 – 7.29 (m, 4H), 7.19 – 7.07 (m, 1H), 6.22 – 6.08 (m, 2H), 5.97 – 5.90 (m, 1H), 4.72 – 4.63 (m, 1H), 4.61 – 4.50 (m, 2H), 4.40 – 4.30 (m, 2H), 3.30 – 3.18 (m, 2H), 3.13 – 3.04 (m, 2H), 2.90 – 2.74 (m, 2H), 2.61 – 2.49 (m, 2H), 2.11 (s, 3H), 1.93 – 1.73 (m, 6H), 1.71 – 1.63 (m, 1H), 1.60 – 1.46 (m, 3H), 1.44 – 1.34 (m, 6H); ^{13}C NMR (126 MHz, $CDCl_3$) δ 191.38, 187.89, 172.55, 155.65, 154.62, 152.75, 149.32, 143.17, 140.95, 139.18, 138.38, 137.42, 136.47, 133.90, 129.65, 129.14, 128.58, 126.92, 126.47, 125.73, 124.89, 120.09, 90.06, 85.04, 75.00, 73.25, 53.55, 51.12, 47.78, 45.56, 37.72, 32.76, 28.78, 26.87, 14.38, 12.87, 12.28; LCMS RT: 2.22 min; MS (*m/z*): $[MH]^+$ calcd. for $C_{41}H_{48}ClN_6O_6S$, 787.3; found, 787.7; Purity (254 nm): 99%.

Supplementary References

1. Manning, G., Whyte, D.B., Martinez, R., Hunter, T. & Sudarsanam, S. The protein kinase complement of the human genome. *Science (New York, N.Y* **298**, 1912-1934 (2002).
