# Supplementary material for: Pharmacological inhibition of PRMT7 links arginine monomethylation to the cellular stress response
Source: Nat Commun. 2020 May 14;11:2396. doi: 10.1038/s41467-020-16271-z (PMC7224190; doi:10.1038/s41467-020-16271-z)
Supplement: Supplementary file 3 — Description of Additional Supplementary Files [file 41467_2020_16271_MOESM3_ESM.docx]

File name:

**Supplementary data 1**.

Description: Kinase selectivity of SGC8158 data relating to Supplementary Fig. 2.

File name:

**Supplementary data 2**.

Description: Identification of monomethyl arginine peptides in PRMT7 WT and KO cells data relating to Fig. 2c and Supplementary Table 4.

File name:

**Supplementary data 3**.

Description: Input peptide level analysis in PRMT7 WT and KO cells data relating to Supplementary Table 4 and Supplementary Fig 5.

File name:

***Source Data***

Description: Source Data for Figs.1-6, Supplementary Figs. 1, 3, 5-15, and Tables 1-3 including all primary data on SGC3027 characterization, HSP70 methylation and the biological significance of PRMT7 driven methylation.
