# Supplementary material for: Pharmacological inhibition of PRMT7 links arginine monomethylation to the cellular stress response
Source: Nat Commun. 2020 May 14;11:2396. doi: 10.1038/s41467-020-16271-z (PMC7224190; doi:10.1038/s41467-020-16271-z)
Supplement: Supplementary file 7 — Reporting Summary [file 41467_2020_16271_MOESM7_ESM.pdf]

nature research

Corresponding author(s): Dalia Barsyte-Lovejoy

Last updated by author(s): Apr 13, 2020

Reporting Summary

Nature Research wishes to improve the reproducibility of the work that we publish. This form provides structure for consistency and transparency in reporting. For further information on Nature Research policies, see Authors & Referees and the Editorial Policy Checklist .

Statistics

For all statistical analyses, confirm that the following items are present in the figure legend, table legend, main text, or Methods section.

n/a	Confirmed	
☐	☒	The exact sample size (*n*) for each experimental group/condition, given as a discrete number and unit of measurement
☐	☒	A statement on whether measurements were taken from distinct samples or whether the same sample was measured repeatedly
☐	☒	The statistical test(s) used AND whether they are one- or two-sided<br>*Only common tests should be described solely by name; describe more complex techniques in the Methods section.*
☒	☐	A description of all covariates tested
☐	☒	A description of any assumptions or corrections, such as tests of normality and adjustment for multiple comparisons
☐	☒	A full description of the statistical parameters including central tendency (e.g. means) or other basic estimates (e.g. regression coefficient) AND variation (e.g. standard deviation) or associated estimates of uncertainty (e.g. confidence intervals)
☐	☒	For null hypothesis testing, the test statistic (e.g. *F*, *t*, *r*) with confidence intervals, effect sizes, degrees of freedom and *P* value noted<br>*Give P values as exact values whenever suitable.*
☒	☐	For Bayesian analysis, information on the choice of priors and Markov chain Monte Carlo settings
☒	☐	For hierarchical and complex designs, identification of the appropriate level for tests and full reporting of outcomes
☒	☐	Estimates of effect sizes (e.g. Cohen's *d*, Pearson's *r*), indicating how they were calculated

Our web collection on statistics for biologists contains articles on many of the points above.

Software and code

Policy information about availability of computer code

Data collection	The MmPRMT7_ SGC8158 dataset was collected at the 24ID-E beamline at the Advanced Photon Source (APS).
Data analysis	The IC50 values and statistical significance (student and multiple t-test, one-way Anova), were determined, where applicable, using GraphPad Prism 7 software. Kinetic curves for SPR analysis were fitted using a 1:1 binding model and the Biacore T200 Evaluation software (Ver.3.1, GE Health Sciences Inc.). Band intensities for western blot analysis were determined using Image Studio Ver 5.2 (Licor). Apoptosis levels and cell confluency were analyzed with IncuCyte™ ZOOM (2015A) software.<br><br>X-ray diffraction dataset was processed with HKL300078. Initial phases were obtained by using MmPRMT7 (PDB ID:4C4A) as initial model in Fourier transform with Refmac5 (version 5 5.8.0238). Model building was performed in COOT (version 0.8.9.2) and the structure was validated with Molprobity (version 5 5.8.0238) . SGC8158 restraints were generated using Grade Web Server (http://grade.globalphasing.org). Images were prepared with PyMol Software (The PyMOL Molecular Graphics System, v2.2.0, Schrödinger, LLC.).<br><br>For intracellular compounds concentration MassLynx 4.1 software from Waters was used for data analysis with the QuanLynx module. Standard curves were generated by using the linear fit of mass peak areas and the known concentrations of SGC8158 and SGC8158N.<br><br>For Mass Spectrometric analysis of arginine monomethylation the raw files were searched and quantified using MaxQuant version 1.6.2.1 and using the UP000005640 UniPprot Release 2018_08 human database (Swiss-Prot reference containing 20,352 protein entries, downloaded on 24 October, 2018). PTM scores for monomethylarginine were generated using the MaxQuant platform as previously described and site level occupancy was calculated by the ratio of modified peptide in two samples, the unmodified peptide version and the protein ratio. P-values from four independent replicates calculated by empirical Bayes moderated t-tests and adjusted using the Benjamini-Hochberg procedure as implemented in the Bioconductor package limma (v3.38.3). Known methylation sites were referenced from PhosphoSitePlus® v6.5.8 for Figure 2c and Supplementary Table 4. Gene ontology enrichment analysis was performed using clusterProfiler (ver. 3.10.1).

For manuscripts utilizing custom algorithms or software that are central to the research but not yet described in published literature, software must be made available to editors/reviewers. We strongly encourage code deposition in a community repository (e.g. GitHub). See the Nature Research guidelines for submitting code & software for further information.

Data

Policy information about availability of data

All manuscripts must include a data availability statement. This statement should provide the following information, where applicable:

- Accession codes, unique identifiers, or web links for publicly available datasets
- A list of figures that have associated raw data
- A description of any restrictions on data availability

All proteomics mass spectrometry data have been deposited in the MassIVE repository with the accession number MSV000084773. The URL to download the data: ftp://massive.ucsd.edu/MSV000084773/. Known methylation sites were referenced from PhosphoSitePlus® v6.5.8 for Figure 2c and Supplementary Table 4. The UP000005640 UniProt Release 2018_08 human database (Swiss-Prot reference containing 20,352 protein entries, downloaded on 24 October, 2018) was used.
Supplementary data 1 file. Kinase selectivity of SGC8158. Data relating to Supplementary Fig 2.
Supplementary data 2 file. Identification of monomethyl arginine peptides in PRMT7 WT and KO cells. Data relating to Fig 2c and Supplementary Table 4.
Supplementary data 3 file. Input peptide level analysis in PRMT7 WT and KO cells. Data relating to Supplementary Table 4 and Suppl Fig 5.
The mPRMT7_SGC8158 structure has been deposited in the PDB (PDB ID: 6OGN). The data is associated with fig 1e,f and Supplementary Figure 4.
Source Data for the Fig1-6 and supplementary Fig 1, 3, 5-15, including the uncropped western blots as well as Tables 1-3 are provided as Source Data file.

Field-specific reporting

Please select the one below that is the best fit for your research. If you are not sure, read the appropriate sections before making your selection.

☒ Life sciences ☐ Behavioural & social sciences ☐ Ecological, evolutionary & environmental sciences

For a reference copy of the document with all sections, see nature.com/documents/nr-reporting-summary-flat.pdf

Life sciences study design

All studies must disclose on these points even when the disclosure is negative.

Sample size	Based on previous experience from similar studies (PMID: 31848333, 31408619, 31285596) and pilot studies, experiments were performed at least 2-3 times to confirm reproducibility. Sample sizes are described in Methods and figure legends.
Data exclusions	Resolution cutoff was applied to the MmPRMT7_SGC8158 X-ray diffraction data using both CC1/2 and (1/delta) pre-established criteria. reference (PMID: 23793146)
Replication	The data were generated from at least 3 technical replicates. In most cases the experiments were successfully repeated on separate dates. All the details are indicated in the manuscript.
Randomization	No specific randomization method was applied. Batches of cultured cells were randomly allocated to treatment groups and control groups.
Blinding	Subsets of X-ray diffraction amplitudes were withheld for calculation of Rfree values.

Reporting for specific materials, systems and methods

We require information from authors about some types of materials, experimental systems and methods used in many studies. Here, indicate whether each material, system or method listed is relevant to your study. If you are not sure if a list item applies to your research, read the appropriate section before selecting a response.

Materials & experimental systems

n/a	Involved in the study	
☐	☒	Antibodies
☐	☒	Eukaryotic cell lines
☒	☐	Palaeontology
☒	☐	Animals and other organisms
☒	☐	Human research participants
☒	☐	Clinical data

Methods

n/a	Involved in the study	
☒	☐	ChIP-seq
☒	☐	Flow cytometry
☒	☐	MRI-based neuroimaging

Antibodies

Antibodies used

Anti-Rme1 (#8015, 1:1000), anti-Rme2s (#13222, 1:2000) and anti-mouse IgG Alexa Fluor 488 (#4408, 1:1000), anti-TIAR (#8509, 1:2000), anti rabbit IgG Alexa647 (#4414, 1:2000) were purchased from Cell Signaling Technologies. Anti-Hsp/Hsc70 was from Enzo (#ADI-SPA-820, 1:2000). Antibodies for PRTMT7 (#ab179822, 1:1000), PRMT5 (#ab109451, 1:5000), H3K79m2 (#ab3594, 1:2000), H4 (#ab174628,1:2000) and β-actin (#ab3280,1:3000) were purchased form Abcam. Anti-PRMT4 (#A300-421A, 1:2000) was from Bethyl. Anti-GFP (#632381, 1:3000) used for western blot was purchased from Clontech. Anti-GFP used for IP was purchased from Invitrogen (#G10362, 1:200). Anti-Flag (#F4799, 1:5000) was from Sigma. Anti-SmBB' (#sc-130670, 1:100) and anti-BAF155 (#sc-32763, 1:200) was from Santa Cruz Biotechnologies. Anti-BAF155-R1064me2a (#ABE1339, 1:3000) was from Millipore. Anti-H4R3me2a (#39705, 1:2000) was from Active Motif. Goat-anti rabbit IgG-IR800 (#926-32211, 1:5000) and donkey anti-mouse IgG-IR680 (#926-68072, 1:5000) were purchased from LiCor. Antibody recognizing methylated SAP145 was kind gift from Dr. Yanzhong Yang, Beckman Research Institute (1:1000).

Validation

We confirmed antibodies: Flag (#F4799, Sigma-Aldrich), GFP (#632381, Clontech) and GFP (#G10362, Invitrogen) by immunoblotting using non-transfected or FLAG or GFP-tagged protein transfected cells. We confirmed antibodies: PRTMT7 (#ab179822, Abcam), PRMT5 (#ab109451, Abcam) and PRMT4 (#A300-421A, Bethyl) by immunoblotting using cells transfected with controlof PRMT7,5 and 4 targeted siRNA, respectively. We confirmed antibodies: Rme1 (#8015, CST) and Rme2s (#13222, CST) by immunoblotting using cells transfected with control or PRMT1,3,4,5,6,7 gene targeted siRNA and with lysates from cells treated with DMSO and selective PRMT1,3,4,5 inhibitors. We confirmed antibody Hsp/Hsc70 (#ADI-SPA-820, Enzo) by immunoblotting using cells transfected with GFP, GFP-HSPA8, 1 and 6 (the antibody recognizes all three proteins). We confirmed antibodies: H4 (#ab174628, Abcam) and BAF155 (#sc-32763, Santa Cruz Biotechnologies) by immunoblotting using cells transfected with GFP, GFP-H4 and GFP-BAF155, respectively. Antibody recognizing methylated SAP145 was validated previously (PMID:25737013). H4R3me2a (#39705, Active Motif), TIAR (#8509, CST), β-actin (#ab3280, Abcam), and SmBB' (#sc-130670, Santa Cruz Biotechnologies) antibodies were validated by suppliers. H3K79m2 (#ab3594, Abcam) and BAF155-R1064me2a (#ABE1339, Millipore) antibodies were validated by suppliers and in previous publications (PMID:23250418 and PMID:27479032, respectively).

Eukaryotic cell lines

Policy information about cell lines

Cell line source(s)

MCF7 (ATCC® HTB-22™), C2C12, MEF WT and MEF Prmt7 KO (kind gift from Dr. Stephane Richard, McGill University), U-2 Os (ATCC®HTB-96™), HT-1080 (ATCC®CCL-121™) and HEK293T (kind gift from Sam Benchimol, York University, ATCC®CRL-3216™), HeLa (ATCC®CRM-CCL-2™), HCT116 WT (ATCC®CCL-247™), THP-1 (kind gift from Dr. Mark Minden, Princess Margaret Cancer Center, ATCC® TIB-202™), MDA-MB-231 (ATCC® HTB-26™). All mammalian cell lines were purchased from Cedarlane and Sf9 cells (#11496015) from ThermoFisher Scientific.

Authentication

All cell lines were tested for authentication by STR profiling.

Mycoplasma contamination

All cell lines were mycoplasma negative, as determined by MycoAlert™ Mycoplasma Detection Kit (Lonza).

Commonly misidentified lines
(See ICLAC register)

No commonly misidentified cell lines were used according to ICLAC database (https://iclac.org/databases/cross-contaminations/)
